# Multiple Geographic Origins of Commensalism and Complex Dispersal History of Black Rats

**DOI:** 10.1371/journal.pone.0026357

**Published:** 2011-11-02

**Authors:** Ken P. Aplin, Hitoshi Suzuki, Alejandro A. Chinen, R. Terry Chesser, José ten Have, Stephen C. Donnellan, Jeremy Austin, Angela Frost, Jean Paul Gonzalez, Vincent Herbreteau, Francois Catzeflis, Julien Soubrier, Yin-Ping Fang, Judith Robins, Elizabeth Matisoo-Smith, Amanda D. S. Bastos, Ibnu Maryanto, Martua H. Sinaga, Christiane Denys, Ronald A. Van Den Bussche, Chris Conroy, Kevin Rowe, Alan Cooper

**Affiliations:** 1 Australian National Wildlife Collection, CSIRO Ecosystem Sciences, Canberra, Australia; 2 Graduate School of Environmental Earth Science, Hokkaido University, Sapporo, Japan; 3 Smithsonian Institution, Division of Birds, Washington D.C., United States of America; 4 Office of the Gene Technology Regulator, Canberra, Australia; 5 South Australian Museum, and Australian Centre for Evolutionary Biology and Biodiversity, University of Adelaide, Adelaide, Australia; 6 Australian Centre for Ancient DNA, University of Adelaide, Adelaide, Australia; 7 Institut de Recherche pour le Developpement, Research Unit 178, Center for Vectors and Vector Diseases, Faculty of Science, Mahidol University, Nakhonpathom, Thailand; 8 Laboratoire de Paléontologie, Phylogénie et Paléobiologie, Institut des Sciences de l'Evolution, Université Montpellier II, Montpellier, France; 9 Department of Biological Resources, National Chiayi University, Chiayi City, Taiwan; 10 Department of Anthropology, University of Auckland, Auckland, New Zealand; 11 Department of Anatomy & Structural Biology, University of Otago, Dunedin, New Zealand; 12 Mammal Research Institute, Department of Zoology & Entomology, University of Pretoria, Pretoria, South Africa; 13 Indonesian Institute of Sciences (LIPI) & Museum Bogoriense, Cibinong, West Java, Indonesia; 14 Department of Systematics and Evolution, Muséum National d'Histoire Naturelle, Paris, France; 15 Department of Zoology, Oklahoma State University, Stillwater, Oklahoma, United States of America; 16 Museum of Vertebrate Zoology, University of California, Berkeley, California, United States of America; Natural History Museum of Denmark, Denmark

## Abstract

The Black Rat (*Rattus rattus*) spread out of Asia to become one of the world's worst agricultural and urban pests, and a reservoir or vector of numerous zoonotic diseases, including the devastating plague. Despite the global scale and inestimable cost of their impacts on both human livelihoods and natural ecosystems, little is known of the global genetic diversity of Black Rats, the timing and directions of their historical dispersals, and the risks associated with contemporary movements. We surveyed mitochondrial DNA of Black Rats collected across their global range as a first step towards obtaining an historical genetic perspective on this socioeconomically important group of rodents. We found a strong phylogeographic pattern with well-differentiated lineages of Black Rats native to South Asia, the Himalayan region, southern Indochina, and northern Indochina to East Asia, and a diversification that probably commenced in the early Middle Pleistocene. We also identified two other currently recognised species of *Rattus* as potential derivatives of a paraphyletic *R. rattus*. Three of the four phylogenetic lineage units within *R. rattus* show clear genetic signatures of major population expansion in prehistoric times, and the distribution of particular haplogroups mirrors archaeologically and historically documented patterns of human dispersal and trade. Commensalism clearly arose multiple times in *R. rattus* and in widely separated geographic regions, and this may account for apparent regionalism in their associated pathogens. Our findings represent an important step towards deeper understanding the complex and influential relationship that has developed between Black Rats and humans, and invite a thorough re-examination of host-pathogen associations among Black Rats.

## Introduction

The Black Rat (*Rattus rattus*; also known as ‘House’, ‘Roof’ and ‘Ship’ Rat) is the most widely distributed of all commensal animals and the most destructive of all animal pests. It is a remarkably adaptable species that plays multiple roles as a household pest [1], a destructive agricultural pest in cereal and vegetable crops, orchards and palm plantations [1], [2], and a feral invader of natural habitats [3], [4]. Huge effort is invested in pest control and feral eradication of the species, and there is growing interest in its role in numerous zoonotic disease cycles, including the ongoing threat posed by plague (*Yersinia pestis*) [5] and the emerging threats posed by bunyaviruses, leptospirosis and a range of bacterial “typhus” syndromes in particular [6], [7].

Despite its obvious socio-economic significance, the Black Rat remains poorly understood from a taxonomic and evolutionary standpoint, and it remains almost entirely unstudied as a wild mammal within its natural range. Studies of chromosomes and blood proteins during the 1960s and 70 s identified patterns of geographic variation in the species [8], and subsequent genetic [9] and morphological work [10] encouraged discrimination of two weakly differentiated species – *R. rattus* for European and Indian populations with a karyotype of 2n = 38–40 and *R. tanezumi* for Asian populations with a 2n = 42 karyotype [11]. However, more recent regional studies of Black Rat mitochondrial DNA (mtDNA) [12]–[13], [14] identified patterns of genetic diversity that are not easily reconciled with this taxonomic arrangement.

We undertook a global survey of mitochondrial DNA (mtDNA) of this important pest animal throughout its entire range. Our findings shed new light on the evolutionary history of Black Rats, including the geographic pattern of diversification and the direction and timing of prehistoric, historic and contemporary dispersals, and suggest a novel explanation for the exceptionally diverse zoonotic disease associations of this particular group of rats. Our findings also point the way forward to resolving the taxonomy of this problematic group of mammals.

## Results

**Figure 1:**
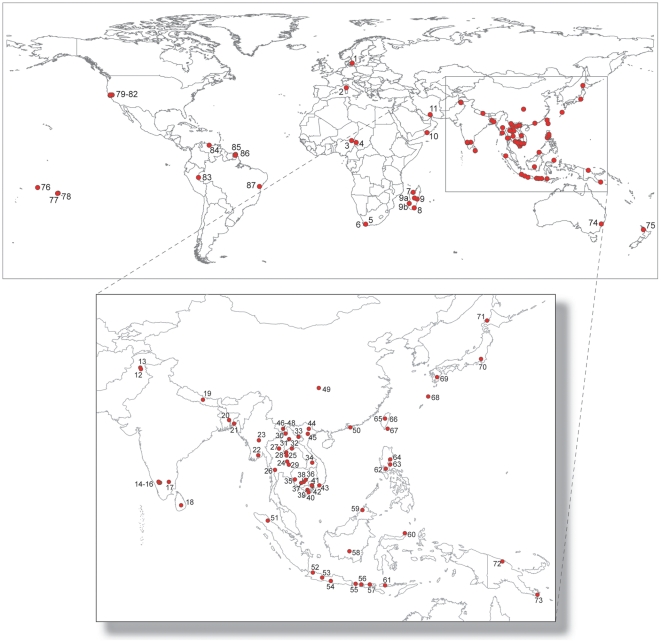
Maps showing the global distribution of sampling localities. Locality numbers refer to entries in Tables S1 and S2.

We obtained mitochondrial *cytochrome b* (*cyt b*) gene sequences from 165 individuals identified on morphological criteria as Black Rats, presumably representing either *R. rattus* or *R. tanezumi*. These were derived from 76 localities in 32 countries (Fig. 1; Tables S1, S2). To test the monophyly of Black Rats and to establish their relationships with other *Rattus*, we obtained *cyt* b sequences from seven other species of *Rattus* and species in four other closely related genera of the Tribe Rattini (Table S1).

### Phylogenetic analysis

**Figure 2:**
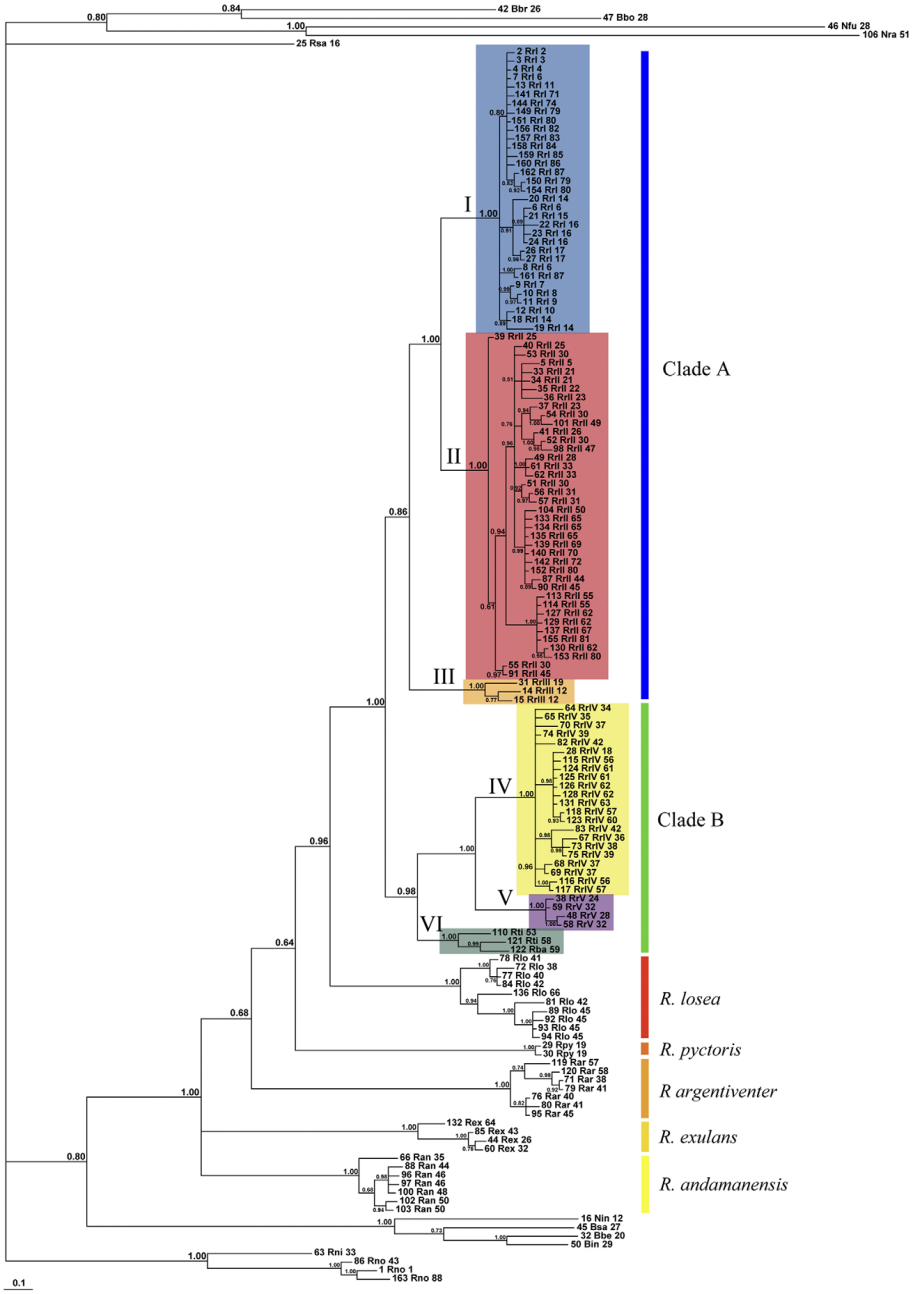
Phylogenetic tree produced with Bayesian Inference using MCMC. The tips are labelled with codes that identify a unique combination of haplotype and locality (i.e. an identical haplotype only appears more than once if it was found at different localities). The full details for each tip code appear in Table S1. This tree also shows relationships outside of the *Rattus rattus* Complex. Numbers on nodes are Bayesian posterior probabilities.

The Black Rat *cyt* b sequences fall into four well-supported lineages (designated I–IV on Fig. 2). Three of these lineages (I–III) comprise an exclusive, monophyletic assemblage of Black Rats designated Clade A on Fig. 2. However, the fourth Black Rat lineage (IV) belongs to a separate clade (designated B on Fig. 2) that otherwise includes rats coming from Thailand and Laos and identified as *R. losea* (Lineage V) and representatives of the Sundaic species *R. tiomanicus* and *R. baluensis*, which together comprise Lineage VI. The term *Rattus rattus* Complex (RrC) [15] is employed here for the smallest monophyletic unit that includes all of the ‘typical’ Black Rats. The mtDNA tree topology suggests that the RrC is polytypic and includes at least four currently recognised species. Table 1 reconciles our mtDNA terminology and taxonomic usage with that employed in several recent studies.

**Table 1:** Correspondence between our mtDNA lineage terminology and taxonomic usage with that employed in several recent studies, along with typical morphology, habitat use and disease associations.

mtDNA lineage	Associated karyotypes	Aplin et al. [15]	Musser and Carleton [11]	Robins et al. [30]	Pages et al. [14]	This Study	Typical morphology	Known habitat Use^5^	Probable disease associations^6^
RrC LI	Oceanian *R. rattus* 2n = 38	*R. rattus*	*R. rattus*	*rattus* clade	R1 = *R. rattus*	*R. rattus* LI	Large, long tailed, scansorial^4^	Secondary forest to urban	BA, CD, LE, LM, PL, RV, TM, WN
RrC LII	Asian *R. rattus* part 2n = 42	*R. tanezumi* northern	*R. tanezumi* part	*tanezumi* clade	R2 = *R. tanezumi*	*R. rattus* LII	Large, long tailed, scansorial	Primary forest to urban	BA?, LE, PL, SC, TY,
RrC LIII	Asian *R. rattus* part ?	Uncategorised	*R. tanezumi* part	Not sampled	Not sampled	*R. rattus* LIII	Medium-sized, long tailed, scansorial	Poorly known; includes urban	?
RrC LIV	Asian *R. rattus* part 2n = 40^1^	*R. tanezumi* southern	*R. tanezumi* part	*diardi* clade	R3	*R. rattus* LIV	Large, long tailed, scansorial	Primary forest to urban	PL?, TY?
RrC LV	*R. losea* part^2^ 2n = 42	*R. losea* western	*R. losea* part	Not sampled	R4 = *R. losea*	*R. sakeratensis*	Small, short tailed, terrestrial	Primary forest to agricultural fields	?
RrC LVI	*R. rattus tiomanicus* 2n = 42	*R. tiomanicus*	*R. tiomanicus*	*R. tiomanicus*	R5 = *R. tiomanicus*	*R. tiomanicus*+*R. baluensis*	Medium to large, long tailed, scansorial	Primary forest, gardens/plantations; occasionally village	?
*R. losea*	*R. losea* part^3^ 2n = 42	*R. losea* eastern	*R. losea* part	Not sampled	Not sampled	*R. losea*	?	?	?

1 A 2n = 40 karyotype was reported by Yosida [8] from specimens referred to *R. kandianus* from the central highlands of Sri Lanka. MtDNA sequences reported here from the same locality are attributable to RrC LIV.

2 Karyotypes of 2n = 42 reported by Yosida [8] and Badenhorst et al. [96] as *R. losea* were obtained from animals from Thailand. MtDNA sequences reported here from the same locality as Yosida's samples are attributable to RrC LV.

3 Karyotypes of 2n = 42 reported by Yu et al. [97] for *R. losea* from Taiwan.

4 Scansorial = able to climb and frequently doing so, but not restricted to arboreal existence.

5 The degree of commensalism of each lineage.

6 Probable disease associations are inferred from geographic distribution of lineages and diseases reported from ‘*Rattus rattus*’. Disease codes: BA: *Bartonella* infections; CD: Chagas Disease; LE: Leptospirosis; LM: Leishmaniasis; PL: Plague; RV: Rift Valley fever; SC: Schistosomiasis; TM: Trypanosomiasis; TY: Typhus (Rickettsial); WN: West Nile Encephalitis. See Table S3, References S1 for further details and referencing of disease associations.

Exemplars of *R. losea* from Cambodia, Vietnam, China, and Taiwan form a sister clade to the RrC. Taiwan is the type locality of *Rattus losea* [16], hence this clade is identified as true *R. losea*. Other recognised species of *Rattus* are represented by monophyletic clades on the *cyt b* tree. At progressively further remove from the RrC are clades representing *Rattus pyctoris*, *R. argentiventer*, *R. exulans* and *R. andamanensis*. Several other species of *Rattus* are even further removed from the RrC, including *R. norvegicus* (the type species of *Rattus*) and *R. satarae* of western India. Tree topology at this level is probably uninformative due to the effects of substitutional saturation. However, the isolated position of *R. satarae*, a taxon that was formerly treated as a subspecies of *R. rattus* [17], is consistent with a published cladistic analysis of LINE-1 retroposons [18] and a more extensive analysis of *cyt* b data [17].

Though our sampling of all other species of *Rattus* is much less extensive than for the Black Rats, our geographic coverage of most species is adequate to suggest that the level of mtDNA divergence between each of the six lineages of the RrC is equivalent to interspecific, rather than intraspecific, differentiation elsewhere in the genus *Rattus* (Fig. 2 and Table 2).

**Table 2:** Total nucleotide divergence (*D_xy_*; above diagonal) and net nucleotide divergences (*D_a_*; below diagonal) between the six lineages of the *Rattus rattus* Complex.

	Lineage I	Lineage II	Lineage III	Lineage IV	Lineage V	Lineage VI
**Lineage I (n = 35)**	-	0.0350±0.0014	0.0468±0.0059	0.0575±0.0029	0.0623±0.0069	0.0500±0.0064
**Lineage II (n = 40)**	0.0283±0.0014	-	0.0478±0.0059	0.0608±0.0029	0.0625±0.0065	0.0493±0.0058
**Lineage III (n = 3)**	0.0338±0.0381	0.0376±0.0061	-	0.0574±0.0090	0.0587±0.0202	0.0503±0.0194
**Lineage IV (n = 22)**	0.0485±0.0506	0.0524±0.0029	0.0472±0.0092	-	0.0359±0.0051	0.0468±0.0074
**Lineage V (n = 4)**	0.0560±0.0585	0.0570±0.0065	0.0514±0.0203	0.0298±0.0306	-	0.0409±0.0142
**Lineage VI (n = 3)**	0.0377±0.0392	0.0368±0.0063	0.0361±0.0196	0.0343±0.0078	0.0314±0.0144	-

All values are calculated with the Jukes-Cantor correction. Number of distinct haplotypes shown in row headings.

#### Divergence times within the RrC

**Figure 3:**
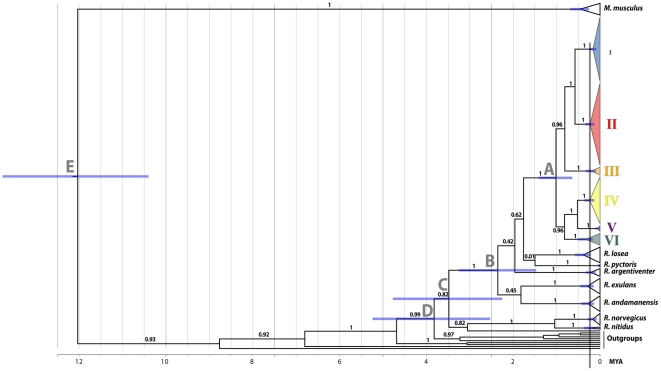
Divergence time estimates for key diversification events in the Rattini and the *Rattus rattus* Complex. Result of BEAST analysis using an uncorrelated lognormal relaxed-clock model and with HKY+I+G6 nucleotide substitution model on data partitioned by the three codon positions. MCMC analyses were run for 30,000,000 steps, with posterior samples drawn every 1000 steps after a burn-in of 3,000,000 steps. The general constant size coalescent model was used as tree prior. Divergence time estimates for labelled nodes A–E are shown in Table S3, References S1.

Molecular clock calculations (Figs. 3, S1; Table S4, References S1) suggest that the RrC lineages initially diverged during the Early to Middle Pleistocene, with a central estimate of 1.015 myr and a 95% probability range of 0.640–1.42 myr. Diversification within each of the four better-sampled RrC lineages is estimated to have commenced around 0.18–0.24 myr (Fig. 3), which broadly overlaps the penultimate interglacial period.

### Biogeographic and phylogeographic patterns within RrC lineages

#### Broad biogeographic patterns

**Figure 4:**
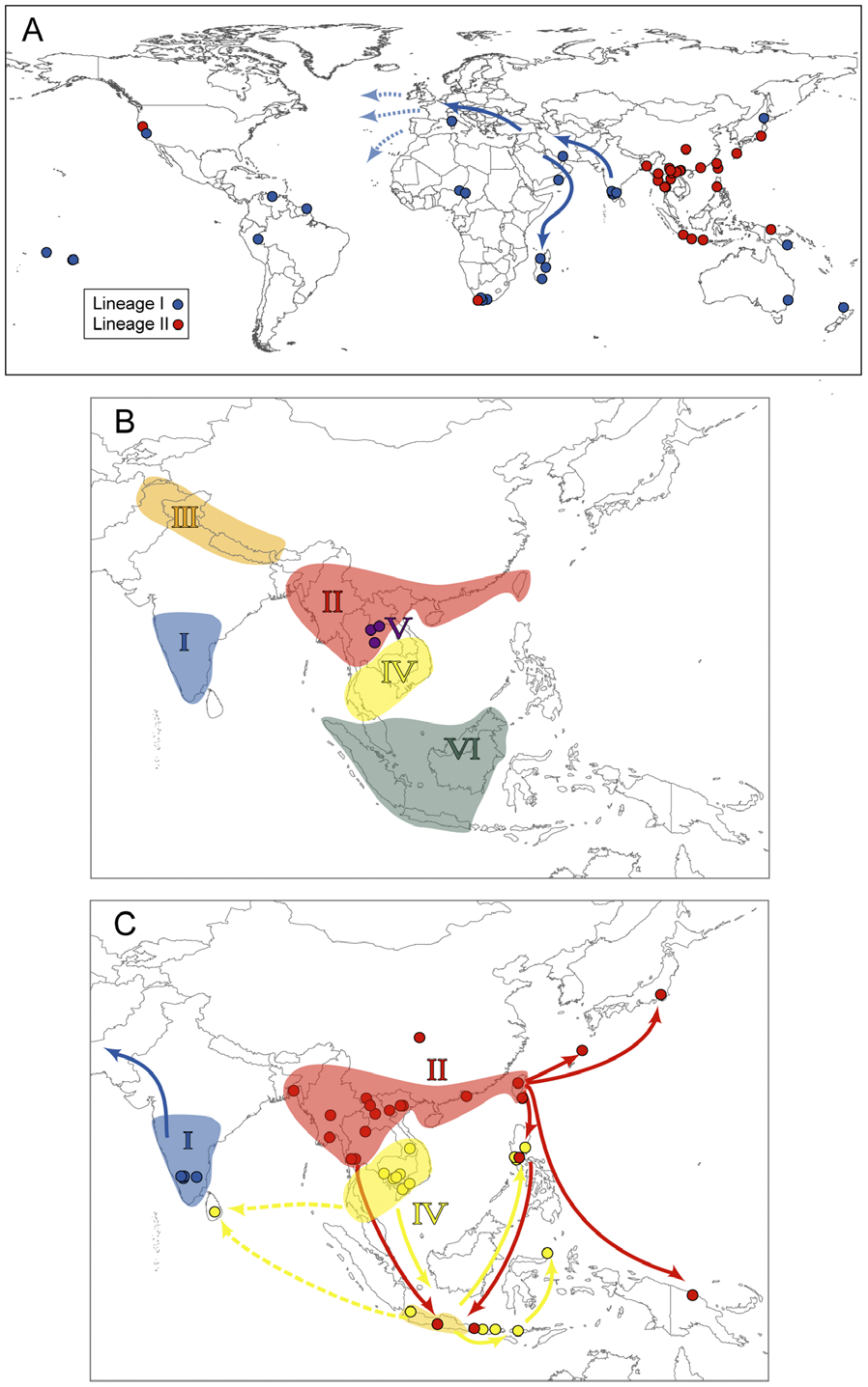
Geographic distribution and inferred dispersal episodes of the six lineages of the RrC. (**A**). Global distribution of lineages I and II, showing inferred direction of movement of lineage I rats into the Middle East and from there, independently to Madagascar and Europe (and thence globally, as ship-borne emigrants). Note that lineage II is represented in South Africa and western USA. (**B**) Semi-schematic diagram showing the inferred natural ranges of each of lineages I–VI of the RrC, including the inferred ‘Sundaic’ sublineages of Lineage IV (hatched), which is fully congruent with the range of Lineage VI. Points of particular interest include: 1) extensive range overlap between lineages II and V (*R. sakeratensis*) in Thailand and central to southern Laos; 2) extensive range overlap between Lineages IV and VI (*R. tiomanicus*) on the Sundaic islands; 3) abutting or narrowly overlapping ranges of Lineages II and IV in central to southern Laos and Thailand; and 4) lack of evidence for natural range overlap among Lineages I–III prior to the onset of widespread habitat disruption and human-mediated dispersal. It is not clear whether the natural range of Lineage II included Taiwan or whether the natural range of lineage I included Sri Lanka. (**C**). Distribution in Asia of lineages I,II and IV showing inferred directions of prehistoric movement for each of lineage. Regional movement of Lineages II and IV has resulted in a broad zone of geographic overlap that includes Indonesia and the Philippines.

RrC Lineages I–IV show geographic distributions on mainland Asia that are, for the most part, mutually exclusive (Fig. 4). Within this geographic realm, Lineage I is present only in western India, Lineage II in eastern India through Myanmar, northern Laos and Vietnam, and southern China, Lineage III in the Himalayan foothills of Pakistan and Nepal, and Lineage IV in the lower Mekong River catchment in southern Laos, Thailand and southern Vietnam. Lineages II and IV overlap on mainland Asia in central Laos and in Thailand [14; as lineages R2 and R3].

Lineage I shows the broadest distribution outside of mainland Asia (Fig. 4), with representation in Europe, the Americas, Africa and Madagascar, and Australia and various Pacific Islands. Lineages II and IV both are widely occurring in the Indo-Malayan region, including the Philippines, while Lineage II is also represented in Japan, Papua New Guinea and South Africa, and in the western part of the USA.

Lineage V was detected only in rats from Thailand and southern to central Laos. It co-occurs with Lineage IV in the south and with Lineage II in the north.

Lineage VI was detected in rats from Java and Borneo, and is represented in exemplars of both *R. tiomanicus* and *R. baluensis* from montane forest on Mt Kinabalu, Sabah. On Java Lineage VI was detected at the same general locality as rats with each of Lineage II and IV.

#### Phylogeographic patterns within Lineage I

**Figure 5:**
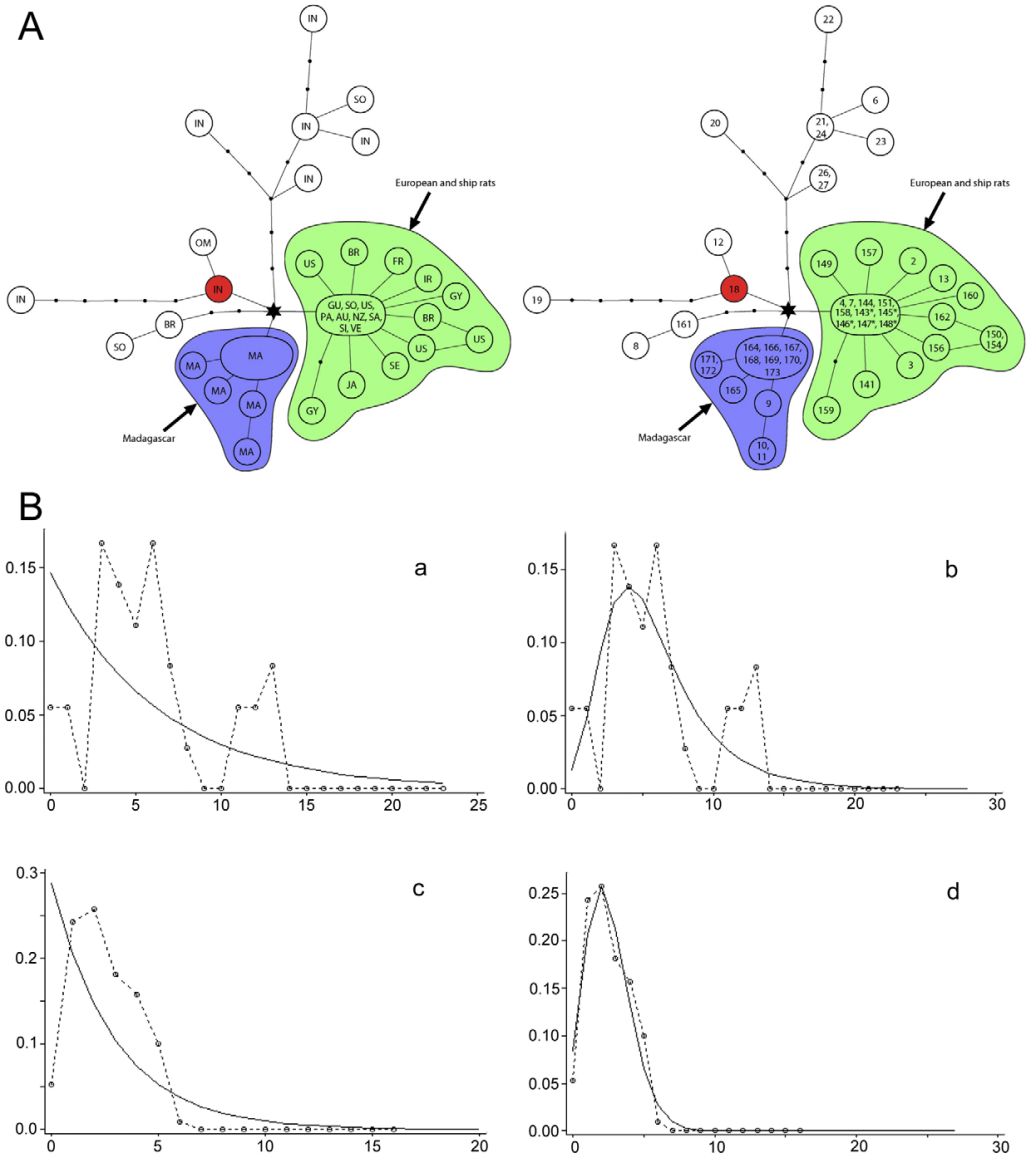
Population genetic analyses of lineage I of the RrC. **A**) Median Joining Networks. On the left network, the observed haplotypes are identified by country of origin (country codes are: AU: Australia; BR: Brazil: FR: France; GU: Guinea; GY: Guyana; IN: India; IR: Iran; JA: Japan; MA: Madagascar; NZ: New Zealand; OM: Oman; PA: Papua New Guinea; SA: Samoa; SE: Senegal; SI: Society Islands; SO: South Africa; US: United States of America; VE: Venezuela); on the right network they are identified by haplotype number as listed in Table S2. In both networks, multiple codes or numbers within a single node signify an identical haplotype coming from more than one country or locality within a country. Haplotypes marked with an asterisk are short sequences and their position on the network was inferred from a separate network analysis that produced an otherwise identical topology; **B**) Pairwise mismatch analyses. Separate analyses were carried out for specimens derived from the inferred natural range of Lineage I on the Indian subcontinent (a–b) and for specimens belonging to the European Black Rat population and its global ‘ship rat’ derivatives (c–d). For each population, the mismatch distribution is compared with curves derived from coalescent simulations under contrasting models of stable (a,c) or fluctuating (b,d) populations.

Lineage I shows its maximum genetic diversity in southern India. The pairwise mismatch distribution for this regional sample (Fig. 5 a–b) shows a ragged pattern typical of long term residency and a stable population, and population genetic statistics confirm this impression (Table 3).

**Table 3:** Population genetic statistics for Lineages I, II and IV and geographic sub-populations of Lineage I and II.

Lineage	N	Nucleotide diversity, *Pi* ± s.d.	Tajima's D	Fu and Li's D*	Fu and Li's F*	Fu's Fs	Ramos-Onsin & Rozas' R_2_
All Lineage I	35	0.0052±0.00054	0.01	0.49	0.50	−0.15	0.11
			−1.65–2.06	−2.40–1.40	−2.65–1.57	−5.17–6.08	0.06–0.18
			n.s.	n.s.	n.s.	n.s.	p<0.01
Lineage I Indian	9	0.0062±0.00143	−0.07	0.49	0.48	0.23	0.17
			−1.69–1.67	−1.99–1.31	−2.11–1.55	−3.56–4.78	0.11–0.26
			n.s.	n.s.	n.s.	n.s.	n.s.
Lineage I ship-rat	21	0.0026±0.00035	0.71	0.47	0.48	0.10	0.14
			0.34–0.89	−2.15–1.38	−2.56–1.57	−3.62–4.56	0.08–0.21
			n.s.	n.s.	n.s.	n.s.	p<0.001
All Lineage II	40	0.0084±0.00058	−0.14	−0.05	−0.08	−0.35	0.11
			−1.75–1.75	−2.30–1.38	−2.58–1.54	−6.36–6.46	0.06–0.17
			n.s.	n.s.	n.s.	n.s.	p<0.05
Lineage II Mainland	22	0.0072±0.00055	−0.11	0.01	0.10	−0.01	0.13
			−1.77–1.73	−2.21–1.36	−2.51–1.55	−4.73–5.70	0.08–0.20
			n.s.	n.s.	n.s.	n.s.	p<0.05
Lineage IIA	10	0.0021±0.00047	0.20	−0.05	−0.07	0.26	0.19
			−1.74–1.85	−1.92–1.38	−2.08–1.58	−2.56–4.19	0.12–0.30
			n.s	n.s	n.s	n.s	p<0.001
Lineage IIB	8	0.0020±0.00038	0.01	−0.04	−0.14	0.32	0.21
			−1.60–1.81	−1.69–1.41	−1.93–1.60	−2.24–3.75	0.14–0.33
			n.s	n.s	n.s	n.s	p<0.01
All Lineage IV	22	0.0084±0.00090	−0.09.	−0.08	−0.15	−0.02	0.13
			−1.75–1.95	−2.58–1.29	−2.86–1.47	−4.89–5.71	0.08–0.19
			n.s	n.s.	n.s.	n.s.	n.s.
Lineage IV Indochina	11	0.0092±0.00116	−0.11	−0.09	−0.09	0.15	0.165
			−1.75–1.69	−2.06–1.36	−2.22–1.48	−3.67–4.67	0.10–0.22
			n.s	n.s	n.s	n.s	p<0.0

N = total number of haplotypes. Values for nucleotide diversity are *Pi* ± s.d. Estimates of Tajima's D, Fu and Li's D*, Fu and Li's F*, Fu's Fs and Ramos-Onsin and Rozas' R_2_ are followed by 95% confidence intervals and statistical significance (n.s. = not significant) derived from 1000 coalescent simulations.

Outside of India, the majority of haplotypes belong to a single star-like cluster with a maximum of two substitutions between the central haplotype and its derivatives. This group is here coined the ‘ship rat’ cluster – it includes individuals from parts of the world in which the introduction of Black Rats is documented historically either during or since the Age of Exploration [19]. Appropriately, it also includes rats from the potential source areas of the middle-east and Europe. A second star-like cluster comprises exclusively haplotypes from Madagascan rats. Each of the ‘ship rat’ and Madagascan clusters are linked to a common haplotype (indicated by a star on Fig. 5A) which in turn is linked to an Indian haplotype (18 on Fig. 5A). Haplotype 18 is here coined the ‘out of India’ haplotype as it appears to represent the ancestral haplotype from which virtually all non-Indian RrC Lineage I haplotypes are derived, including one found exclusively in a rat from Oman (haplotype 12 on Fig. 5A).

Two Lineage I haplotypes (6,8 on Fig. 5A) detected in South Africa and one detected in Brazil (161 on Fig. 5A) fall outside of the ‘ship rat’ cluster. Haplotypes 8 and 161 differ from each other by a single base pair substitution, while haplotype 161 differs by two substitutions from the ‘out of India’ haplotype. Haplotype 6 from South Africa is derived from Indian haplotypes 21/24 and represents an independent emigrant lineage. The *tau* value of 2.49 (Table 4) for the ‘ship rat’ cluster indicates a comparatively recent population expansion but the minimum estimate of time since the start of this expansion (using the highest suggested *cyt* b substitution rate and the shortest generation time) still falls around 4000 years ago. Unless *cyt* b mutation rates are not appreciably different over periods of 10^2^ and 10^3^ years [20], this suggests that haplotype diversity within the ‘ship rat’ cluster is inherited from a genetically diverse European source population. This is readily testable through wider geographic sampling of extant European Black Rats.

**Table 4:** Expansion time estimates for geographic sub-populations of Lineages I, II and IV.

	tau	Mutation rate		k	generation time		expansion time estimate (ybp)			
Lineage		*u1*	*U2*		GT1	GT2	u1+GT1	u1+GT2	u2+GT1	u2+GT2
Lineage I Indian	3.052	2.3	4	945	0.3	1	21063	70209	12111	40370
Lineage I ‘ship rat’	2.486	2.3	4	945	0.3	1	17157	57189	9865	32884
Lineage II Mainland	6.766	2.3	4	945	0.3	1	46694	155648	26849	89497
Sublineage IIA	1.956	2.3	4	945	0.3	1	13500	45000	7762	25900
Sublineage IIB	1.857	2.3	4	945	0.3	1	12800	42700	7369	24600
Lineage IV Indochina	6.49	2.3	4	945	0.3	1	44790	149298	25754	85847

	tau	Mutation rate		k	generation time		expansion time estimate (ybp)			
Lineage		*u3*	*U4*		GT1	GT2	u3+GT1	u3+GT2	u4+GT1	u4+GT2
Lineage I Indian	3.052	6	10	945	0.3	1	8074	26914	4844	16148
Lineage I ‘ship rat’	2.486	6	10	945	0.3	1	6577	21922	3946	13153
Lineage II Mainland	6.766	6	10	945	0.3	1	17899	59665	10740	35799
Sublineage IIA	1.956	6	10	945	0.3	1	5170	17200	3104	10300
Sublineage IIB	1.857	6	10	945	0.3	1	4910	16400	2947	9830
Lineage IV Indoochina	6.49	6	10	945	0.3	1	17169	57231	10302	34339

Estimates of *tau* ( = expansion time in mutational units) were obtained from coalescent simulations as implemented in DnaSP version 5.10.1 [77]. These were converted into years before present (ybp) with the formula ybp = generation time (GT) multiplied by tau/2*u*k (where *u* = mutation rate per site per year and k = sequence length; [83]). We used four different estimates of *u* (all shown as 10×10^−8^), drawn from studies of murine rodents in general [98]–[99] or from analysis of the *Rattus* Division [100], and two values for generation length (0.3 and 1.0, the former being the shortest time to first breeding in *Rattus rattus* [101] and the latter representing a situation where breeding occurs on a seasonal basis and only once each year). The four values of *u* are derived as follows: *u1* = 3^rd^ codon position substitutions only, estimate based on results from arvicoline and murine rodents [98]; *u2* = lower estimate of substitution rate for all positions within the *Rattus* Division [100], based on origin of the genus *Rattus* at 3.0 mya (maximum age suggested by murid-wide analysis of two nuclear genes: *IRBP* and *RAG1*); *u3* = upper estimate of substitution rate for all positions within the *Rattus* Division [100], based on origin of the genus *Rattus* at 2.0 mya (minimum age suggested by murid-wide analysis of *IRBP* and *RAG1* genes and fossil record); *u4* = estimate of silent divergence rate from comparative analysis of mammalian sequences [99]. The combination of values for each parameter produces eight expansion time estimates for each lineage. The expansion time model u4+GT1, using the most rapid mutation rate (10×10^−8^ per site per year) and the shortest generation time (0.3 yr), produced values closest to expectation based on archaeological and historical evidence for the time of major dispersal events. The very wide range in estimates of mutation rate reflect intense saturation of the *cyt* b gene at quite low levels of divergence, even when silent substitutions alone are considered.

#### Phylogeographic patterns within Lineage II

**Figure 6:**
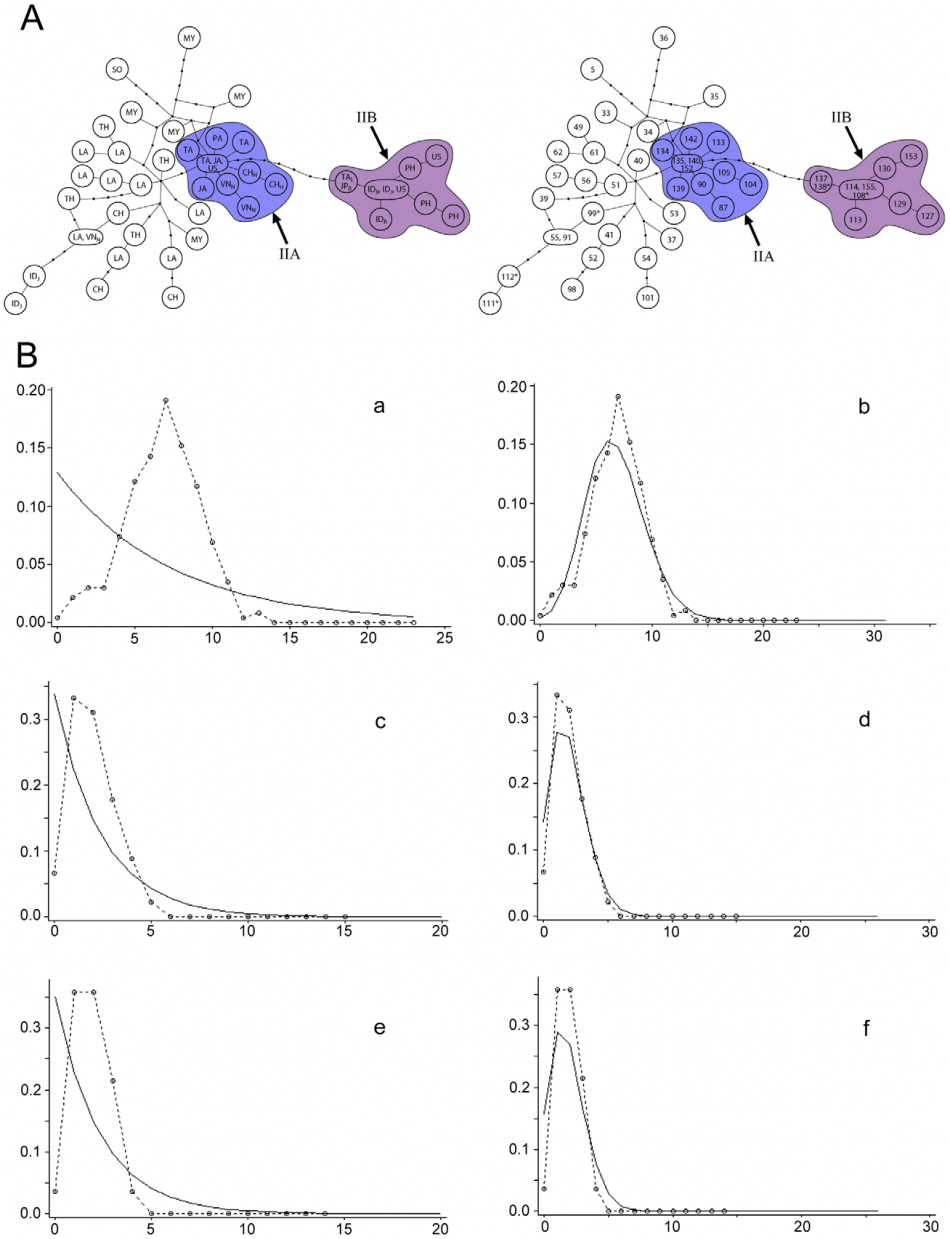
Population genetic analyses of lineage II of the RrC. **A**) Median Joining Network constructed as for Fig. 5. In the left network the observed haplotypes are identified by country of origin Country (codes as follows: BA: Bangladesh; CH: mainland China; CH_H_: Hong Kong; ID_B_: Indonesia (Bali Island) ; ID_J_: Indonesia (Java Island); JA: Japan; JA_R_: Japan (Ryukyu Archipelago); LA: Laos; MY: Myanmar; PA: Papua New Guinea; PH: Philippines; SO: South Africa; TA: Taiwan; TA_L_: Taiwan [Lanyu, Orchid Island]); TH: Thailand; US: United States of America; VE: Venezuela; VN_N_: Vietnam (northern); in the right network they are identified by haplotype number as listed in Table S1. In both cases, multiple codes or numbers within a single node signifies an identical haplotype coming from more than one country or locality within a country. Haplotypes marked with an asterisk are short sequences and their position on the network was inferred from a separate TCS analysis that produced an otherwise identical topology. **B**) Pairwise mismatch analyses, calculated as for Fig. 5. Separate analyses were carried out for specimens derived from the inferred natural range of lineage II on the Indochinese mainland (a–b) and for specimens belonging to each of sub-lineages IIA (c–d) and IIB (e–f) that derive mainly from areas of the western Pacific where archaeological evidence independently documents recent prehistoric introductions. For each population, the mismatch distribution is compared with curves derived from coalescent simulations under contrasting models of stable (a) or fluctuating (b) populations.

Lineage II shows its highest local haplotype and nucleotide diversity in populations from mainland Indo-China (Laos, Myanmar, and Thailand). The pairwise mismatch distribution of mainland populations of Lineage II shows a unimodal pattern suggestive of recent population expansion rather than a ragged pattern indicative of long term population stability (Fig. 6B a–b). However, the *tau* estimate of 6.77 (Table 4) suggests a considerably more ancient population expansion than that of the ‘ship rats’ of Lineage I. Estimates of the time since expansion vary between 10,740 years and 155,600 years (Table S4, References S1), with the lower estimates probably more realistic if the results for the ‘ship rat’ lineage are any guide.

Two peripheral star-like clusters are present in the Lineage II network (labelled IIA and IIB of Fig. 6). The distribution of both clusters is centred on the western Pacific region. Cluster IIA mainly comprises haplotypes from the western Pacific margin, including Taiwan, Japan, coastal China (Hong Kong) and northern Vietnam (near Hanoi). The central haplotype of Cluster IIA is represented in rats from Taiwan and also from Japan and the United States, and differs by three substitutions from haplotypes from Thailand and Laos. Cluster IIB comprises haplotypes from Lanyu (Orchid Island), southwest of Taiwan, Luzon Island in the Philippines, Anami-oshima Island in the Ryukyu Archipelago, Bali and Java Islands in Indonesia, and from the United States. Cluster IIB appears to be derived from the central haplotype of Cluster IIA but the connecting branch is very long, involving eight substitutions. The central haplotype of Cluster IIB is represented in rats from Indonesia and the United States.

Lineage II haplotypes detected in rats from South Africa (6 on Fig. 6A) and Java, Indonesia (111, 112 on Fig. 6A) fall outside of these clusters and are peripheral to different regions of the network.

#### Phylogeographic patterns within Lineage IV

Lineage IV is represented by fewer sequences than either Lineages I or II but shows equivalently high nucleotide diversity (Table S5, References S1). Although the network is less densely populated it is nevertheless clear that Lineage IV shows its highest local haplotype and nucleotide diversity in populations located in the lower catchment of the Mekong River. The pairwise mismatch distribution for these mainland Indochinese populations is ragged, typical of a stable population, but population genetic statistics support a hypothesis involving some degree of population growth (Table 3). As with the mainland population of Lineage II, the estimate of *tau* is quite high (6.49; Table 4), indicating an ancient phase of population growth.

**Figure 7:**
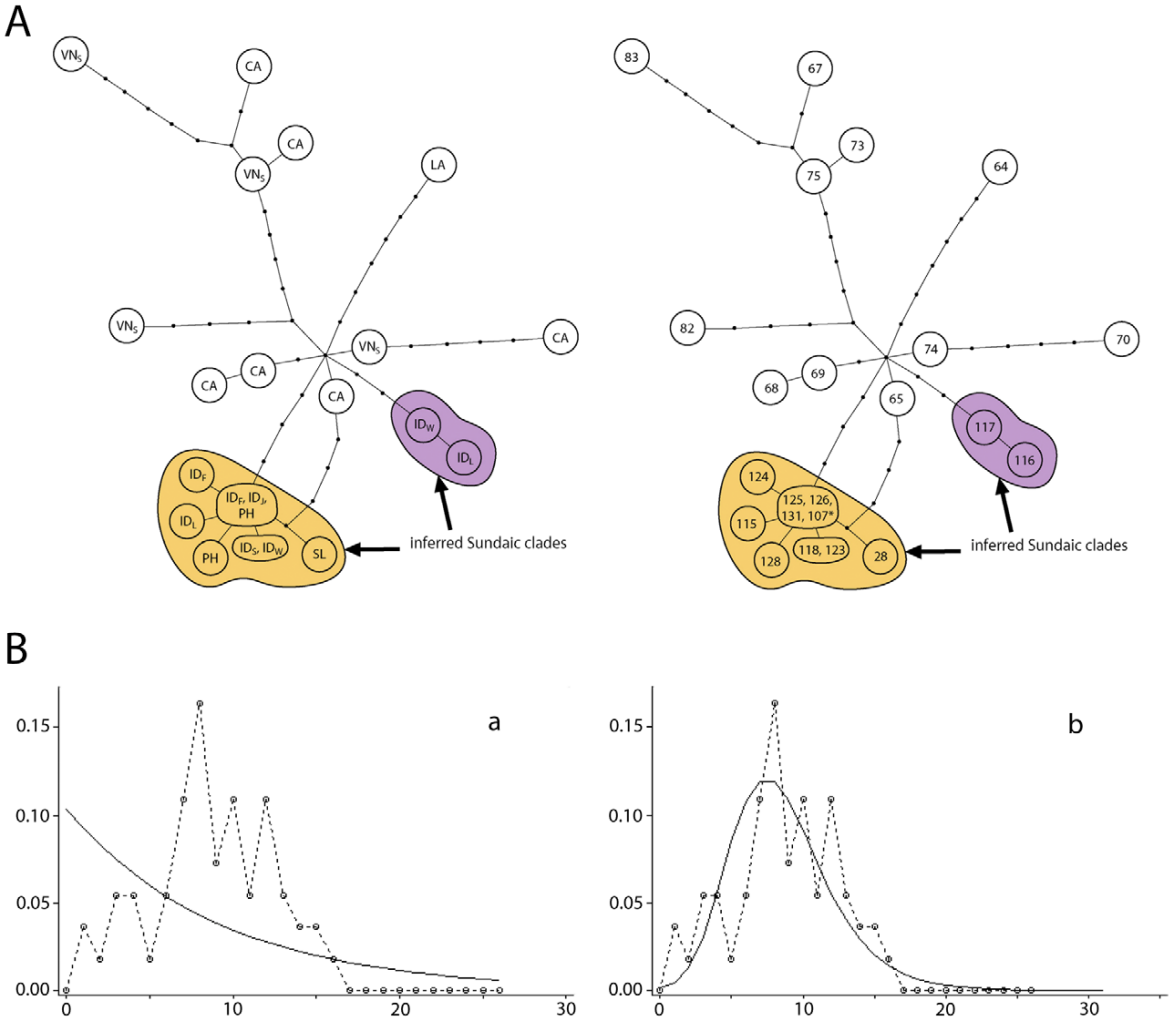
Population genetic analyses of lineage IV of the RrC. **A**) Median Joining Network, constructed as for Fig. 5. In the left network the observed haplotypes are identified by country of origin [Country codes CA: Cambodia; ID_F_: Indonesia (Flores Island); ID_L_: Indonesia (Lombok Island); ID_J_: Indonesia (Java); ID_S_: Indonesia (Sulawesi); ID_W_: Indonesia (Sumbawa Island); LA: Laos; PH: Philippines; SL: Sri Lanka; VN_S_: Vietnam (southern)]; in the right network they are identified by haplotype number as listed in Table S1. In both cases, multiple codes or numbers within a single node signifies an identical haplotype coming from more than one country or locality within a country. Haplotypes marked with an asterisk are short sequences and their position on the network was inferred from a separate TCS analysis that produced an otherwise identical topology. **B**) Pairwise mismatch analyses for mainland Indochinese samples of Lineage IV, calculated as for Fig. 5. The inferred Sundaic sublineages are excluded from this analysis because the majority derive from islands where archaeological evidence independently documents recent prehistoric introductions. The mismatch distribution is compared with curves derived from coalescent simulations under contrasting models of stable (a) or fluctuating (b) populations.

All Lineage IV rats from eastern Indonesia and the Philippines fall into one of two peripheral branches (labelled “inferred Sundaic clades' on Fig. 7), one of which has a star-like pattern with a central haplotype represented in rats from Java, Flores and the Philippines, and derivative haplotypes differing by one or two substitutions. Although only a few of these samples actually derive from the continental ‘Sundaic’ islands, the remainder come from oceanic island groups that are peripheral to the Sunda Shelf and which are likely to have received their Black Rats in recent prehistoric times (see Discussion). As argued above for the ship rat cluster, much of the diversity within these sub-lineages probably reflects their yet to be sampled presence on the main Sundaic islands of Java, Borneo and Sumatra.

The discrete nature of the Sundaic sub-lineages and their and peripheral position on the network suggests comparatively recent establishment of these populations on the Sunda Shelf, and the mutual exclusivity of the mainland and Sundaic populations on the network suggests limited subsequent exchange of Lineage IV haplotypes between the Sundaic islands and mainland Indochina.

A haplotype detected thus far only in rats from the Sri Lankan highlands is positioned somewhat ambiguously on the network, either as a derivative of the ‘Sundaic’ cluster or as an independent derivative of a Cambodian haplotype (65 on Fig. 7A).

#### Notes on Lineages V and VI

To help clarify the taxonomic identity of Lineage V we obtained from the National Museum Sweden, Stockholm, a small piece of skin with hair attached from the lectotype of *Rattus sakeratensis* Gyldenstolpe, 1917, a puppet skin collected at Sakerat in eastern Thailand. This taxon is currently treated as a synonym of *R. losea* [16], [21] and is the earliest available name for ‘*R. losea*’ from Indochina. By using aDNA methods we generated good quality sequence for a 437 bp section of *cyt* b and a Bayesian inference phylogenetic analysis resulted in strongly supported assignment of the resultant haplotype within Lineage V.

Within Lineage VI, a haplotype obtained from a specimen of *R. baluensis* from Mt Kinabalu, Sabah is nested between haplotypes derived from *R. tiomanicus* from Java and Kalimantan.

### Phenotypic and ecological differentiation

Rats with mtDNA Lineages I–IV are all superficially alike, with tails that equal or exceed the length of the body, feet that are moderately broad, and prominent plantar pads. These features are consistent with the scansorial lifestyle of Black Rats. Dorsal and ventral pelage coloration is highly variable within populations carrying each of mtDNA Lineages I, II and IV, with the dorsal pelage showing a range of reddish to brown hues peppered with black, and the ventral fur variably pure white to cream, or grey-based but bearing cream to buff or greyish tipping. Melanistic individuals with grey to black fur typically show no differentiation in dorsal and ventral fur colour. Cranial and dental morphology is variable between populations but it is not clear whether or not the variation between rats with contrasting mtDNA lineages exceeds that observed within single lineages. Detailed morphometric analyses are currently underway to further explore this variability.

Rats with Lineages I–IV all can be found as commensal populations as well as pests in agricultural fields. Rats with Lineages II and IV are also known from natural forest habitats in Southeast Asia where they tend to be comparatively scarce except in areas of recent disturbance.

Rats with mtDNA Lineage V are consistently smaller than the others and have narrower hind feet and shorter tails than any of the typical Black Rats. A more detailed morphological description of this taxon is available from accounts in which they were treated as a regional variant of *R. losea* [16], [21]. Field studies of RrC Lineage V rats in Cambodia indicate that they are non-commensal but common in agricultural fields [22].

Rats with mtDNA Lineage VI are similar in body form to the typical Black Rats but most have greyer dorsal pelage, usually combined with a cream ventral fur. These rats are consistent with descriptions of *R. tiomanicus* [21], [23]; these accounts should be consulted for a detailed account of external and cranio-dental differentiation from typical Black Rats. *Rattus tiomanicus* is common in secondary forest and plantations, and less so close to human habitation [21].

## Discussion

Our study provides the first global perspective on the evolutionary history of Black Rats and their close relatives. Though many gaps remain in our geographic coverage, our sampling in the key ancestral region of South and Southeast Asia is sufficiently dense to support a new hypothesis on the evolutionary origin and diversification of *Rattus rattus*. Additionally, our findings yield key insights into the complex dispersal history within the group, and provide new perspectives on the origin of commensalism in the group and on the diverse zoonotic disease associations of Black Rats. Our results also clarify the taxonomic identity of the short-tailed Indochinese rats currently but erroneously included within *R. losea*.

### Evolutionary history and taxonomy of the *Rattus rattus* Complex

The mtDNA gene tree presented here portrays the RrC as one element of geographically dispersed but comparatively recent radiation within the genus *Rattus* that includes a number of other species with Indochinese (e.g. *R. andamanensis*, *R. argentiventer*, *R. losea*), Himalayan (*R. pyctoris*), and Indo-Malayan distributions (*R. exulans*). The Norway Rat (*R. norvegicus*), the type species of the genus *Rattus*, appears to be phyletically more remote, as are several less well-known species of *Rattus* (e.g. *R. satarae* of western India).

Our molecular clock estimates of initial diversification of *Rattus* at 3.5 mya and for the origin of the RrC at 1.0 mya are consistent with other recent estimates based on small numbers of whole mtDNA genomes [24] and multiple unlinked genes [25]–[27] and with the earliest occurrence of fossils attributable to the RrC in deposits of middle Pleistocene age in Thailand [28] and Java [29]. The Javan deposits, dated to around 1 million years ago, contain two possible members of this group, one with morphological affinities with *R. rattus*, the other closer to *R. tiomanicus*.

The six primary mtDNA lineages within the RrC show only partial correspondence either with currently recognised *Rattus* species or with chromosomally defined ‘races’ of *R. rattus*. Lineage I does show good fidelity with populations commonly identified as *R. rattus*, including rats from European and Indian localities, and with the 2n = 38 ‘Oceanian’ karyotype of Yosida [9]. By contrast, populations with Lineages II, III and IV would all be regarded as *R. tanezumi* under current taxonomy. Most populations associated with these lineages have 2n = 42 karyotypes (‘Asian’ of Yosida's terminology) but one population of Lineage IV on Sri Lanka has 2n = 40 [8]. Himalayan populations sampled by Yosida [8] have 2n = 42 but the mtDNA affinity of the karyotyped rats is unknown.

Lineage IV was detected by each of Robins et al. [30], who suggested a possible association with *R. rattus diardii*, and Pages et al. [14], who considered it to be a novel taxon based on attributes of tree topology as well as regional sympatry with Lineage II in Thailand. Our results confirm those of Pages et al. [14] in suggesting a closer relationship of Lineage IV with several other currently recognised species of *Rattus*, represented in our study by Lineages V and VI.

Lineage V corresponds with cluster R4 of Pages et al. [14] who identified it as *R. losea*. Unlike all previous assessments, our study included exemplars of true *R. losea* from the type locality of Taiwan, as well as from multiple localities in southern China, northern and southern Vietnam, and Cambodia. These populations cluster separately from the Thai and Laotian rats of Lineage V, and fall outside of the RrC as defined here. Morphological comparisons, to be presented elsewhere, also highlight the distinction between the Thai/Laotian populations and true *R. losea* and favour recognition of Lineage V as a distinct species. The nomenclature of this previously unsuspected member of the genus *Rattus* is resolved in the following section. Morphological and ecological similarity between this taxon and true *R. losea* might be due to retained ancestral ecomorphology or to a parallel terrestrial adaptation. The pattern of ecomorphological evolution within the genus will become clear once details of *Rattus* phylogeny are clarified by wider genome sampling.

Lineage VI includes *R. tiomanicus* of the Malay Peninsula and the Sundaic islands, and *R. baluensis*, a montane endemic of northwestern Borneo. *Rattus tiomanicus* is broadly co-distributed with ‘typical’ Black Rats across a large geographic area and displays a consistent morphological segregation [23]. It inhabits both forest and disturbed habitats but, unlike the Black Rats, generally avoids villages and towns. The restricted montane endemic *R. baluensis* is phenotypically well-differentiated from each of *R. tiomanicus* and the Black Rats [23]. Our result suggests that *R. baluensis* may have arisen by local adaptive divergence or drift from within the more widespread *R. tiomanicus*. However, other interpretations are possible, most notably that *R. tiomanicus* mtDNA has introgressed into *R. baluensis*. The correct scenario will be decided by closer examination of the genetic history of these groups on a regional to local scale.

Paraphyly of the ‘typical’ Black Rats is an intriguing prospect but whether or not the mtDNA trees accurately portray the evolutionary history of the RrC requires confirmation with additional, unlinked genes. An alternative scenario that needs to be excluded is ancient introgression into a population of Black Rats in the Lower Mekong catchment or Sundaic area from a now extinct Clade B mitochondrial lineage. However, any such event would need to predate diversification within Lineage IV, which is estimated to have commenced around 0.25 myr ago. Nuclear gene trees that show well-supported monophyly of Lineage IV with the other Black Rats would favour this history.

Leaving aside this issue of paraphyly, the deep and geographically highly structured mitochondrial diversity among the Black Rats suggests an ancient range expansion that resulted in broad colonisation of mainland Indochina and South Asia, followed by long history of allopatric divergence of at least partially isolated populations. Based on patterns of haplotype and nucleotide diversity, the likely geographic focus of diversification within each lineage was: eastern and southern India for Lineage I; the western part of Indochina and uplands of eastern Indochina for Lineage II; the Himalayan foothills for Lineage III; and the lower Mekong River catchment for Lineage IV. These geographic populations probably remained at least partially isolated, at least to mtDNA diffusion, until the emergence of human agricultural communities and later still, regional civilizations, provided Black Rats with the opportunity for local, regional, and eventually, global dispersal.

The current taxonomic arrangement of the Black Rats into two species (*R. rattus* and *R. tanezumi*) does not rest easily atop this model of multi-regional differentiation. However, the model itself suggests a way toward taxonomic rationalization. As commonly acknowledged by evolutionary biologists [31], allopatric divergence is probably the most common route to speciation among higher vertebrates, yet paradoxically, it is allopatric populations that generate most taxonomic uncertainty and dispute. The core issue is whether allopatric populations are sufficiently differentiated to warrant recognition at either subspecies or species level. If spatial isolation of the populations is absolute, the decision may need to be an arbitrary one, at best guided by some ‘yardstick’ of acceptable intra-populational variation [32]. However, in many cases, secondary contact occurs either in natural or artificial contexts, and while this may not replicate natural interaction, it does provide an opportunity to assess the strength of reproductive isolation. Possible scenarios include unhindered gene flow (indicating no effective postzygotic isolation; often taken as evidence for lack of speciation); limited introgression involving parts of the genome only, with establishment of a stable or gradually fluctuating hybrid zone (variably taken as grounds for species or subspecies recognition); and establishment of sympatry or parapatry without significant gene flow (indicative of species level differentiation). In the absence of natural secondary contact, laboratory crosses or other kinds of artificial contact provide useful clues to possible biological outcomes.

In the case of the Black Rats, the location of possible natural contact zones between mtDNA lineages is not yet known. However, there is a significant body of information available on laboratory crosses; and the complex history of human-assisted dispersals has produced a number of instances of artificial secondary contact. Laboratory crossing experiments have focussed on the potential post-zygotic barriers generated by various chromosomal rearrangements [7]. Crosses among rats with 2n = 38 (Lineage I), 2n = 42 (Lineage II) and 2n = 40 from Sri Lanka (Lineage IV) karyotypes all produced viable F_1_ hybrids that reached sexual maturity. However, F_2_ hybrids were produced with difficulty in all cross combinations except for the 2n = 38 and 2n = 40 (Sri Lanka) pairing.

Yosida [8] identified a few localities where multiple chromosomal races had come into secondary contact. These were identified initially through recognition of hybrid karyotypes and in one case, investigated further through serology and allozyme analysis. More recently, instances of multiple introductions involving Lineages I and II have been reported in Japan [12], [33], in South Africa [34], on the west coast of the United States [this paper], and in Australia [35]–[36]. In the cases where data that can used to assess gene flow was obtained, the analyses suggest a moderate to high level of gene flow among the immigrant populations, though perhaps with variable outcomes.

In South Africa, Black Rats with mtDNA Lineages I and II generally have 38 and 42 chromosomes, respectively, though two possible F1 hybrids with 2n = 40 were observed in a series of eight individuals with Lineage I *cyt* b haplotypes [34]. Similar fidelity between chromosomal races and mtDNA lineages was reported by Chinen et al. [12] in the case of a recently introduced population of Lineage I rats with 2n = 38 in a predominantly 2n = 42 Lineage II stronghold in northern Hokkaido, Japan. However, Chinen et al. [12] reported evidence for wider introgression of nuclear genes associated with Lineage I rats, perhaps due to an earlier, undocumented introduction. Kambe et al. [33] also identified likely introgression of Lineage I genetic material in Japan, involving the nuclear *Mc1r* gene.

Taking into account all available lines of evidence, we advocate an abandonment of the current bipartite separation of *R. tanezumi* from *R. rattus*. However, because we suspect that each of the four allopatric populations of Black Rats will prove to have significant descriptive utility and will ultimately be accorded some taxonomic recognition (if only as subspecies), we recommend the use of an informal taxonomy that recognizes each of the four naturally occurring allopatric populations according to their mtDNA lineage; i.e. as *Rattus rattus* Lineages I–IV. This is preferable to attempting to fix species or subspecies names at this point in time for each of the regional forms, due to the need for resolution of various nomenclatural issues including the status of various early names (e.g. *Mus asiaticus* Gray, 1837; *Mus indicus* Desmarest, 1822) and the genetic identity of early name-bearing specimens from localities where a history of multiple introductions and genetic admixture is likely (e.g. Sri Lanka). Furthermore, we caution that significant introgression may have occurred, even within parts of the ancestral areas of each lineage, such that the mtDNA identity of an individual or even an entire population might not reflect its dominant genetic identity. Broad genome sampling will be required to clarify the extent of these problems and ultimately lay the foundation for a more stable taxonomy of the *Rattus rattus* Complex.

### The identity of RrC Lineage V

There appears to be only one available name for the taxon represented by mtDNA Lineage V. *Rattus sakeratensis* Gyldenstolpe, 1917 (type locality: Sakerat, eastern Thailand) was erroneously described on a study skin of a *Rattus* mismatched with a skull of *Maxomys whiteheadi*. Marshall [21] designated the skin only as the lectotype and noted its similarity to regional populations referred to *R. losea* (Swinhoe, 1871). One other available name for a member of the RrC comes from within the known or inferred range of Lineage V is *Rattus rattus thai* Kloss, 1917 (type locality: Raheng, central Thailand), but this is a larger, longer-tailed animal that is more likely associated with RrC Lineage II or IV.

The type locality of *Mus losea* Swinhoe, 1871 is Taiwan. Musser and Newcomb [16] noted slight differences in external and cranial morphology between the regional sample from Indochina and samples of typical *R. losea* from Taiwan and adjacent parts of China, and our studies (to be presented elsewhere) confirm this distinction. *Rattus rattus exiguus* Howell, 1927, described from Yenpingfu, Fukien Province, China, is regarded as a synonym of *losea* [16], [21].

A skin and hair sample from the lectotype of *R. sakeratensis* (Royal Natural History Museum, Stockholm, Mammals 560932) produced a partial *cyt* b sequence (GenBank JN812337) that clusters unambiguously with Lineage V of the RrC. While this result is consistent with Marshall's morphological assessment of the lectotype as a specimen of ‘*R. losea*’, caution is required over several points: 1) potential contamination of the lectotype with mtDNA from another specimen of *R. sakeratensis*, either in the host museum or the laboratory; 2) potential retention of an ancestral allele; and 3) potential genetic exchange between the lectotype individual of *R. sakeratensis* and another naturally co-occurring population of *Rattus*.

DNA contamination of the lectotype seems unlikely as there was no possible source for an appropriate contaminant. The lectotype was the only example of this taxon collected by Gyldenstolpe in Thailand [37] and the National Museum of Sweden holds no other collections that might include other examples of *R. sakeratensis*. The skin sample provided for our study remained in a sealed container until it was opened within the ancient DNA facility. Although other samples of *Rattus* had been processed in the ancient DNA facility, these did not include any other specimens of Lineage V of the RrC. Accordingly, we have full confidence that the extracted mtDNA derived from the skin sample.

The reciprocal monophyly of cyt *b* lineages within the RrC clearly argues against retention of an ancestral allele. However, genetic exchange between Lineage V and a different species of *Rattus* cannot be entirely ruled out as a possibility. However, two points argue against hybridization or genetic introgression. The first is the agreement between the morphological and genetic assessment of the specimen. Small body size, a short tail relative to body length, and relative small narrow feet are some of the features that distinguish rats of Lineage V from most other species of *Rattus* found in central Thailand (i.e. *R. rattus* and *R. argentiventer*, *R. andamanensis*), while larger body size, a non-spinous pelage and larger feet distinguish Lineage V rats from *R. exulans* [15], [21]. The second is the fact that specimens identified on morphological criteria as ‘*R. losea*’ from Thailand and Laos have thus far yielded only Lineage V mtDNA, and conversely, no rats with Lineage V have displayed a contrasting morphology. Thus, at present, there is no evidence of mtDNA exchange between rats with mtDNA Lineage V and other, regionally occurring *Rattus* species.

Based on this evidence, we identify *R. sakeratensis* Gyldenstolpe, 1917 as the earliest available name for Lineage V of the RrC and as a valid species distinct from *R. losea* and the ‘typical’ Black Rats.

### Dispersal and range expansion among the Black Rats

Previous treatments of Black Rat dispersal have focused on the presumed migration of rats from India to Europe [38], the historically documented global spread of ship rats [19], and the prehistoric to recent dispersal of rats in the Western Indian Ocean [39]–[40]. The pattern of mitochondrial variation revealed by our global study clearly depicts each of these events. Additionally, however, it reveals a number of previously undocumented aspects of population growth and range expansion among Black Rats, the majority of which have occurred exclusively with the Asian realm.

#### Prehistoric to modern dispersals of Lineage I rats

Apart from a somewhat anomalous record of *R. rattus* from middle Pleistocene cave sediments of Turkish Thrace [41], the earliest evidence of *R. rattus* in Eurasia comes from the Levant [42], possibly dated to 15,000 BP or younger [38]. The earliest Mediterranean records are of pre-Roman age, c. 6000 BP [38] and by the 4^th^ Century, the roof rat was in Britain [43]). The palaeontological and archaeological records of the Middle East and Europe suggest a possible late Pleistocene migration of Black Rats from India to the Middle East. The mtDNA evidence supports this general notion but allows further inferences to be drawn regarding the dispersal process. Remarkably, with the exception of a few haplotypes found in South Africa and Brazil, all other Lineage I haplotypes detected outside of western India appear to be derived from a single emigrant haplotype (number 18 on Fig. 5) which we have dubbed the ‘out of India’ haplotype. Three independent offshoots of this haplotype are recorded and all are derived through single base pair substitutions. One is recorded only in Oman; another gave rise to a star-like radiation in Madagascar; and a third produced the ‘ship rat’ cluster which is presumed to represent as yet an otherwise unsampled local radiation in Europe. Tollenaere et al.'s [40] study of *R. rattus* in the Western Indian Ocean shows many of these same features including the divergent ship rat and Madagascan clusters (labelled Groups C and B, respectively) and the close association of Group C with a particular Indian haplotype (labelled Hap4). However, their study provides an important additional detail, namely the presence in Oman of multiple highly divergent haplotypes, suggesting either a long period of residency of Lineage I on the Arabian Peninsula or multiple dispersals across the Arabian Sea. The absence of shared haplotypes between their Indian and Oman samples favour the former interpretation but sampling in both areas is probably inadequate to rule out either scenarios.

The earliest archaeological evidence of *R. rattus* in Madagascar dates to around the 10^th^ Century [44] but the presence of an *in situ* radiation with similar nucleotide diversity to that found in the ‘ship rat’ cluster clearly favours a longer history, perhaps even associated with the earliest human migrations to Madagascar about 2300 yr BP [45]. Tollenaere et al. [40] estimated the onset of population expansion in the immigrant Madagascan Black Rats at around 3000 yr BP which is consistent with this scenario and the notion that the environment was rapidly transformed following human arrival.

The ‘ship rat’ cluster is a striking feature of the mitochondrial gene topology and it gives new resonance to previous reports of low global mitochondrial diversity among historically introduced populations of *R. rattus* [5], [40]. Given the history of ship-borne dispersal of Black Rats during the Age of Exploration, the limited diversity within this group is surely a reflection of low variation within European source populations, and a strong indication that Black Rats arrived in Europe only during the Neolithic or Bronze Age [43], [46].

South African Lineage I haplotypes include typical ‘ship rats’ as well as several others that fall outside of this restricted cluster (Fig. 5) [34]. These may represent independent dispersal events that occurred direct from the Middle East or India, possibly with early Arab trading activities, as suggested previously on morphological grounds [47]. Alternatively, they might represent otherwise undetected components of ‘ship rat’ genetic diversity, possibly coming out one or more restrict source areas in Europe.

Ship-borne dispersal of Lineage I rats presumably continues today but in many contexts it probably goes undetected. Suzuki et al. [48] documented the arrival of Lineage I Black Rats at a sea port on Hokkaido island, Japan, in a context where the dominant mtDNA is Lineage II. However, studies of Japanese rats using nuclear markers point to low level contamination of the Japanese Black Rat population by genetic components derived from earlier invasions of Lineage I rats [12], [33]. The occurrence of non-‘ship rat’ Lineage I haplotypes in Brazil and South Africa is suggestive of independent, ship-borne translocations of Black Rats out of India or the Middle East.

#### Prehistoric to modern range expansions and dispersals of Lineage II and IV rats

Population genetic analyses of Lineage II haplotypes points to a phase of population growth on mainland Asia during the terminal Pleistocene or early Holocene. Palynological evidence records a widespread transition at this time from broadly distributed pine and oak forests to a more complex mosaic of broadleaf evergreen and deciduous plant communities over much of Thailand [49] and southern China [50], with indications of regular burning in several areas, possibly due to human activities. These conditions of environmental instability may have favoured a disturbance specialist and allowed Black Rats to assume a greater dominance in small mammal communities. This may have also facilitated geographic range expansion, although the relatively high haplotype and nucleotide diversity recorded across much of mainland Indochina suggests a general pattern of local population growth within an originally large geographic range, rather than dramatic range expansion into formerly unoccupied territory. The emergence and spread of agricultural activities with the resultant modification of natural into anthropogenic habitats is likely to have further advantaged populations of Black Rats and provided a context for the development of close associations between human and rat populations. Lineage IV shows a similar signal of population expansion but remains too poorly sampled within its likely natural range to infer further details of population history.

Islands off the continental shelf of East and Southeast Asia evidently lacked any form of Black Rat prior to their arrival in the mid- to late Holocene. Rats are convincingly absent from Japanese fossil assemblages of late Pleistocene age but are present in archaeological samples of late Holocene age [51]. Similar evidence of Holocene introductions is repeated in the fossil and archaeological record of oceanic islands in eastern Indonesia, e.g. Sulawesi [29], Flores [52]–[53] and Timor [28], [54] and in wider Melanesia [55]–[57], with the earliest evidence for *R. rattus* both in Timor and in Micronesia dating to around 3000 BP [28], [54]–[55]. The mtDNA data provide a clear signal of prehistoric dispersal of Black Rats into the western Pacific region during the mid- to late Holocene. The most likely source for dispersal of Lineage II rats, based on the distribution of ancestral genotypes, is Taiwan or southern China/northern Indochina. The few available Taiwanese haplotypes are diverse and distinct from those recorded from mainland localities and it seems likely that Lineage II occurred naturally on Taiwan, which had intermittent land connection to mainland China through the Quaternary [58].

Two sub-lineages of Lineage II were carried into the near Pacific including Japan and the Philippines, and probably from the latter, to the islands of eastern Indonesia and Micronesia. The inferred dispersal of these sub-lineages Lineage II rats parallels the diaspora of Austronesian-speakers out of Taiwan around 4,000 years ago and the associated spread of agricultural lifestyles throughout the Philippines, Indonesia, Micronesia and beyond, into the remote Pacific [59]. Much remains to be learned regarding the routes and timing of dispersal but it is noteworthy that Black Rats are recorded archaeologically in Micronesia, near the geographic terminus of their journey, at ca. 3000 BP [60]. Possible back-migration to coastal areas of mainland Asia, such as northern Vietnam and Hong Kong Island, is also indicated by the network structure of sub-lineage IIA.

Approximately synchronous dispersal occurred among several sub-lineages of Lineage IV. The homeland in this instance appears to be the Lower Mekong Basin of mainland Indochina, or possibly the Malay Peninsula which remains unsampled. Somewhat surprisingly, the Sundaic island of Java appears to lack ancient haplotype diversity in Lineage IV and it seems likely that this island did not support a native population of Black Rats, perhaps because of competition in natural forest habitat with *R. tiomanicus*.

Two emigrant sub-lineages of IV are represented in the islands of Indonesia but only one of these is also known from the Philippines. A dispersal route that involved initial colonisation of the Sundaic and Wallacean islands seems likely, with later northward movement into the Philippines (Fig. 4c). The lack of any haplotype sharing between mainland Indochina and the Indo-Malayan islands suggests either limited opportunities for dispersal between these areas following initial establishment, or a strong local competitive deterrent to back migration.

A Lineage IV haplotype, detected in the Sri Lankan highlands, represents the soft-furred *kandianus* form of earlier taxonomies. The geographic location of this haplotype is not altogether unexpected given the presence in the 4^th^ Century of outposts of the ancient Ceylonese Hindu empire on the islands of Java and Bali [61]. However, ambiguity in the haplotype network also leaves open the possibility of dispersal direct from mainland Indochina (Fig. 4c).

Recent historical periods saw comparatively little dispersal among Asian rats to match the global spread of the European-derived ‘ship rats’. Nevertheless, a number of historical or even contemporary translocations of Lineages II and IV have come to light through recent genetic screening, including occurrences of single haplotypes of Lineage II in South Africa, in California, and in Australia. This most recent phase of Black Rat dispersal is clearly ongoing and is likely to produce increasingly intermingled global distributions of the four commensal Black Rat lineages.

### Multiple origins of commensalism and its implications for zoonotic diseases

Exactly when and how commensalism arose is not well-documented for any of the small mammals that have adopted this way of life. A recent discussion of this issue for Black Rats postulated a unique origin of commensalism within a population resident on the Indian subcontinent, perhaps associated with a genetic innovation in this population [38]. Our results suggest a very different scenario in which commensalism arose multiple times and in different geographic populations of Black Rats. This scenario has important implications for human history as well as for interpretation of host–pathogen co-evolution in this group of mammals.

Two observations are critical in inferring that commensalism arose multiple times among the Black Rats. First, as amply demonstrated by the BEAST analysis, mtDNA lineage diversification among Black Rats is far more ancient than the late Pleistocene to early Holocene emergence of agriculture or any form of settled human lifestyle in Asia or elsewhere [59], [62]–[63]. These contrasting time frames are readily accounted for under a model of multiple origins of commensalism. By contrast, a single origin hypothesis would require either that commensalism represents an ancestral condition in the group (a nonsensical proposition), or that it evolved recently but was subsequently shared among the sampled populations through gene flow or ‘cultural’ transmission. The latter scenarios warrant consideration but are at odds with the evidence presented here for well-maintained phylogeographic structuring in the homeland of the RrC on mainland Asia.

We postulate that commensalism arose many times among geographically dispersed populations of Black Rats. Furthermore, we suggest that this reflects a pre-adaption as disturbance specialists. Across Indochina, Black Rats of lineages II and IV are uncommon in primary forest except in areas of local disturbance, such as along river banks. In contrast, they are the dominant rodent species in all disturbed and artificial habitats within the human-modified landscape [15], [64]. The success of Black Rats in disturbed contexts is readily explained by their exceptionally high reproductive potential (a product of short gestation, large litter size, and short period of post-natal dependency), and their high phenotypic and behavioural plasticity [1]. Throughout much of Asia today, Black Rats are not obligate commensals but rather they exploit the entire human landscape of villages, field complexes, and disturbed forests [64]. Whether commensal populations show any genetic modification relative to their wild relatives is not known. However, we might anticipate a long history of selection for genetic variants that improved survival and reproduction of individual rats living in and around agricultural settlements, possibly including socio-behavioural traits of the kind documented between commensal and non-commensal populations of house mice [65]. Several authors have noted the tendency for rural Black Rats populations to be grey-bellied rather than white-bellied, thereby distinguishing them from the majority of forest and field inhabiting populations.

Zoonotic diseases associated with Black Rats are remarkable not only for their diversity but also for their strong but largely unremarked geographic patterning (Tables 1 and S1). Our findings cast new light on both issues. As circumscribed here, the natural range of the RrC encompasses four of the six sub-regions of the Indo-Malayan zoogeographic realm – the Indian, Himalayan, Indochinese, and Sundaic sub-regions – each of which hosts a distinctive mammalian fauna [66]. Many pathogens detected in species of *Rattus* have been acquired through host switching from co-occurring groups of wild mammals [67]–[68], and rats living in different zoogeographic region thus are likely to have acquired regionally distinctive suites of pathogens, leading to a higher than average pathogen diversity among Black Rats as a whole. The separate origin of commensal Black Rat populations in each region, in turn, could be expected to produce regionally contrasting suites of zoonotic diseases. Future studies of rat-borne zoonoses clearly need to include genetic typing of the host animals as a high priority. For some classic disease studies, this may be possible retrospectively, providing rat populations remain unchanged. From a historical perspective, our findings also invite reconsideration of the biological context of various disease epidemics. For example, we note here that each of the three plague pandemics are believed to have emerged out of regions occupied by different lineages of Black Rats (the Justinian Plague from Africa – lineage I as immigrants; the 14^th^ Century Black Death from central Asia – most likely within the range of lineage III; and the 19^th^ Century Pandemic from East Asia – the heartland of lineage II [69]).

### Future research directions

Our findings prompt numerous other questions regarding the history and biology of the RrC. For example, do the genetically differentiated regional populations of Black Rats have contrasting impacts on agricultural systems and natural ecosystems in different parts of the world? And do subtle biological differences between the lineages result in different susceptibility to management tools currently in use, including major classes of rodenticide? Finally, the known capacity of at least some members of the RrC to interbreed [8] raises the question of whether populations of mixed ancestry might possess biological properties different from those of parental lineages.

While the mitochondrial genetic framework introduced here represents an important first step towards deeper understanding the complex and influential relationship that has developed over millennia between various populations of Black Rats and humans, exploration of the diversity in the nuclear genome and broader genetic interactions among members of the RrC will also need to be articulated before the evolutionary history of Black Rats is written in its full complexity, and with ongoing dispersal and novel interactions a high likelihood, new chapters in this history may still be in the making.

## Materials and Methods

### Ethics Statement

Sample collecting by KPA was carried out under CSIRO AEC Permit No: 00/01 – 28 titled “Taxonomic studies of agricultural pest rodents and their relatives in Southeast Asia”. All other samples were sourced from existing tissue collections associated with museum collections.

### Sample and locus selection

Our sampling of Black Rats focussed on the Asian region which is widely regarded as the place of origin of both the genus *Rattus* and of Black Rats specifically [8]. However, we also attempted to include samples from all continents, and from major island groups (Table S2). In Asia, we sampled populations in rural contexts and in remnant forest habitats rather than in major towns to maximize our chances of sampling native lineages or those derived from prehistoric introductions. Populations in larger towns and ports are more likely to be of mixed origin due to introductions in modern times.

Sampling coverage in Southeast Asia includes one significant gap spanning the Malay Peninsula and it is also sparse for the larger Sundaic Islands (Sumatra, Borneo). It is similarly sparse for the Indian subcontinent but adequate for southern India where chromosomal diversity is greatest [8]. Our sampling of Europe is also very incomplete. However, because of the strong historical evidence for dispersal of European ‘ship rats’ during the past 500 years or less [19], [38], we anticipated that our sampling in Africa, the Americas and Australia would be representative of diversity within the secondary ‘source area’ of Europe.

We included samples of a variety of other species of *Rattus* including some that are considered to be close relatives of *R. rattus* [11]. For these potentially related species, we attempted to sample multiple individuals from across the species' entire geographic range so as to establish a benchmark for typical levels of phylogeographic diversity within *Rattus*.

The mitochondrial *cytochrome* b gene (*cyt* b) was selected for analysis because: 1) mitochondrial DNA has proven utility for revealing phylogeographic structure within mammalian taxa [70] as well as dispersal histories, including those associated with human translocation [71]; 2) a prior study of Japanese Black Rats [12] found an appropriate level of variation in *cyt* b between two karyomorphs; and 3) we have access to a large *cyt* b dataset for *Rattus* and related murines of the Tribe Rattini [25].

### Molecular methods

The majority of sequences were determined from liver samples obtained from modern specimens. A small number of sequences were obtained from skin and hair samples removed from museum vouchers (see below). The following primer combinations were used for particular DNA amplifications: A) mcytb1: CCA TCG TTG TAA TTC AAC TAT AG, mcytbL400: CAT GAG GAC AAA TAT CAT TCT GAG G, mcytbHb: GAA TGG GAG AAT GAA GTG GAA TGC G, mcytb4: CTT TGG CTT ACA AGA CCA AGG TAA; B) L14724: TGA YAT GAA AAA YCA TCG TTG, H15915: CAT TTC AGG TTT ACA AGA C; C) L14724: 5′ CGA AGC TTG ATA TGA AAA ACC ATC GTT G 3′, HRa1025: 5′ GGG TGT TCT ACT GGT TGG CCT CC 3′.

The *cyt* b gene was sequenced for more than 200 individuals (Table S1), either in entirety or in part, depending on the quality of available tissues. A small number of sequences were obtained from GenBank. Most sequences included a common alignment of 945 bp and this was used for the primary analysis of 153 sequences providing unique combinations of haplotype and locality. A subsequent round of analyses used a shorter alignment of 596 bp to accommodate a further 14 sequences. Details of collecting localities are given in Table S3, References S1.

We designed several sets of primers to amplify overlapping smaller fragments of the *cyt* b gene from museum vouchers of *Rattus*. Primers were designed from an alignment of six *Rattus* species and human *cyt* b – where feasible, primers included nucleotides that mismatched human sequences at the 3′ end of each primer to exclude amplification of human contamination. The six reference sequences are: *Rattus praetor* DQ191487; *Rattus tanezumi* DQ191488; *Rattus exulans* DQ191486; *Rattus everetti* DQ191485; *Rattus rattus* AB033702; and *Rattus norvegicus* AB033713. Predicted PCR product lengths were (including primers): 235 bp for RattuscytbF2 5′ TCA TCA GTT ACC CAC ATC TGC 3′ and RattuscytbR2 3′-ACC CTA GTC GAA TGA ATC TGA GG-5′; and 306 bp for RattuscytbF1 5′ATC ACA CCC TCT ACT CAA AA 3′ and RattuscytbR1 5′CTA ATY CGA TAC TTA CAT GCC 3′.

DNA was extracted from small (1–2 mm^2^) pieces of dried skin using Qiagen DNeasy tissue kit as per the manufacturer's instructions. Control extracts and PCR negative controls were included. We used Invitrogen's Platinum Taq Hi Fidelity (MgSO_4_, buffer and enzyme) employing 50 cycles of amplification for PCR. Products of expected size were observed on agarose gels in the sample lanes, all control lanes were clean. PCR products were cleaned using magnetic cleanup system (Agencourt, Ampure) and sequenced using the same primers as for the PCR using ABI Big Dye 3.1 chemistry. The majority of sequences were determined from liver samples obtained from modern specimens. A small number of sequences were obtained from skin and hair samples removed from museum vouchers (see below). The following primer combinations were used for particular DNA amplifications: A) mcytb1: CCA TCG TTG TAA TTC AAC TAT AG, mcytbL400: CAT GAG GAC AAA TAT CAT TCT GAG G, mcytbHb: GAA TGG GAG AAT GAA GTG GAA TGC G, mcytb4: CTT TGG CTT ACA AGA CCA AGG TAA; B) L14724: TGA YAT GAA AAA YCA TCG TTG, H15915: CAT TTC AGG TTT ACA AGA C; C) L14724: 5′ CGA AGC TTG ATA TGA AAA ACC ATC GTT G 3′, HRa1025: 5′ GGG TGT TCT ACT GGT TGG CCT CC 3′.

The *cyt* b gene was sequenced for more than 200 individuals (Table S1), either in entirety or in part, depending on the quality of available tissues. A small number of sequences were obtained from GenBank. Most sequences included a common alignment of 945 bp and this was used for the primary analysis of 153 sequences providing unique combinations of haplotype and locality. A subsequent round of analyses used a shorter alignment of 596 bp to accommodate a further 14 sequences. Details of collecting localities are given in Table S3, References S1.

We designed several sets of primers to amplify overlapping smaller fragments of the *cyt* b gene from museum vouchers of *Rattus*. Primers were designed from an alignment of six *Rattus* species and human *cyt* b – where feasible, primers included nucleotides that mismatched human sequences at the 3′ end of each primer to exclude amplification of human contamination. The six reference sequences are: *Rattus praetor* DQ191487; *Rattus tanezumi* DQ191488; *Rattus exulans* DQ191486; *Rattus everetti* DQ191485; *Rattus rattus* AB033702; and *Rattus norvegicus* AB033713. Predicted PCR product lengths were (including primers): 235 bp for RattuscytbF2 5′ TCA TCA GTT ACC CAC ATC TGC 3′ and RattuscytbR2 3′-ACC CTA GTC GAA TGA ATC TGA GG-5′; and 306 bp for RattuscytbF1 5′ATC ACA CCC TCT ACT CAA AA 3′ and RattuscytbR1 5′CTA ATY CGA TAC TTA CAT GCC 3′.

DNA was extracted from very small (1–2 mm^2^) pieces of dried skin using the Qiagen DNeasy tissue kit as per the manufacturer's instructions. Control extracts and PCR negative controls were included. We used Invitrogen's Platinum Taq Hi Fidelity (MgSO_4_, buffer and enzyme) employing 50 cycles of amplification for PCR. Products of expected size were observed on agarose gels in the sample lanes, all control lanes were clean. PCR products were cleaned using magnetic cleanup system (Agencourt, Ampure) and sequenced using the same primers as for the PCR using ABI Big Dye 3.1 chemistry. Authenticity of *cyt* b sequences obtained from museum vouchers was controlled and assessed using a number of standard ancient DNA criteria, including conducting all pre-PCR work (DNA extraction and PCR set-up) in a physically remote ancient DNA laboratory employing positive HEPA-filtered air pressure, bleach and UV decontamination and full PPE; including negative extraction controls and PCR negative controls alongside all sample extractions and PCR amplifications; intra-laboratory replication of all sequences from independent PCRs; and visual assessment of nucleotide substitutions to ensure they conform with molecular evolutionary expectations (ts>tv, 3^rd^ codon>1^st^ codon>2^nd^ codon substitutions, synonymous>non-synonymous substitutions, absence of intra-sequence stop codons).

### Phylogenetic and Population Genetic analyses

Phylogenetic analyses were performed on manually aligned sequences under Bayesian Inference (BI) using MrBayes version 3.1.2 [72], and the Neighbour Joining (NJ) Method and Maximum Parsimony (MP) as implemented in MEGA version 3.1 [73]. Appropriate models of DNA evolution were determined using ModelTest [74]. For the BI analysis, MCMC were run for 5×10^6^ generations with each codon position as a separate partition with the (GTR+I+Γ) model of nucleotide substitution. Convergence was assessed from multiple chains and by ensuring that effective sample sizes for parameters were >200. Visual inspection of a plot of log likelihood against generation was use to select ‘burnin’ trees to discard prior to summarising the search results with a majority rule consensus tree. For NJ and MP methods we performed 1000 pseudoreplicates to obtain estimates of nodal non-parametric bootstrap support. The tree generated under BI is shown in Fig. 2.

For each of the analyses, we included representatives of various other species of *Rattus* (*R. andamanensis*, *R. argentiventer*, *R. exulans*, *R. losea*, *R. nitidus*, *R. norvegicus*, *R. satarae*) with the following objectives: 1) to test the monophyly or otherwise of *R. rattus*; 2) to identify the closest relatives of *R. rattus* within *Rattus*; and 3) to compare nucleotide diversity within *R. rattus* with the inter- and intra-specific nucleotide diversity in each species of *Rattus* and its close relative *Bandicota*. We also included representatives of other genera of the Rattini (*Berylmys* and *Niviventer* [14]) to serve as outgroups.

The phylogenetic structure of better sampled sub-lineages was investigated using Median-Joining Network analyses [75] and the inferential methods developed in comparable studies of human dispersal history [76]. To quantify diversity and test for population expansions, we calculated a variety of population genetic parameters and statistics for different mtDNA lineages and geographic groupings (Table 3), using DnaSP version 5.10.1 [77]. Specifically, we calculated nucleotide diversity *Pi*, Tajima's D [78], Fu and Li's D* and F* [79], Fu's Fs [80], and Ramos-Onsin and Rozas's R_2_ [81]. We also produced graphs of pairwise mismatch distribution [82], using the coalescent simulation approach implemented in DnaSP version 5.10.1 [77] (Figs. 5, 6, 7). Estimates of *tau* ( = expansion time in mutational units) were converted into years before present (ybp) with the formula ybp = generation length multiplied by tau/2*u*k, where *u* = substitution rate per site per year; k = sequence length [83]. We used various prior estimates of *u*, drawn from studies of murine rodents in general or from analysis of the *Rattus* Division (see Table 4). For each of lineages I and II, separate analyses were carried out on populations within the inferred natural range *vs* populations resulting from range expansion into areas not originally occupied by any member of the Black Rat group.

Where the karyotype of individual rats included in this study is known from prior studies, this information is included in Table S1. No karyotypes were determined specifically for our study.

### Molecular clock analyses

*Cytochrome b* sequences reported by Robins *et al.* [24] were added to the current dataset to enable further comparison of results. Six House Mouse (*Mus musculus*) *cytochrome* b sequences (GenBank accession numbers EF108340-5) were added to incorporate a calibration point from the murine fossil record. The widely used divergence of *Mus* and *Rattus* actually corresponds to the origin of tribal diversity within mainland Asian representatives of the subfamily Murinae [25]. The best substitution model was selected using ModelGenerator by comparison of Akaike Information Criterion 2 scores [84]. Bayesian phylogenetic analysis with timescale was performed with BEAST version 1.4 [85], using the divergence time estimation of 10.4–14 Mya [64] for the *Mus*/*Rattus* node. An uncorrelated lognormal relaxed-clock model [86] was used with HKY+I+G6 substitution model on the data partitioned into the three codon positions. MCMC analyses were run for 30,000,000 steps, with posterior samples drawn every 1000 steps after a burn-in of 3,000,000 steps.

Due to the complex mixture of intra and inter-specific diversity in the dataset, the general constant size coalescent model was chosen as tree prior (see Fig. 3). However, applying a general model will not always conciliate such different levels of genetic diversity. In order to check for any bias in time estimations due to this issue, a similar BEAST analysis was conducted using only one representative per clade (these are identified in Table S4, References S1), and applying the Yule process speciation model as tree prior (see Fig. S1). Comparison of the two sets of results does not indicate significant differences in estimated dates according to 95% margins (Table S4, References S1). The BEAST input files are available from the authors upon request.

Robins *et al.* [24] calculated the tMRCA for four clades that are present in our data (see Table S4, References S1), using whole mitochondrial sequences and a narrower calibration date range (*Mus*/*Rattus*, 11–12.3 Mya [87]). We defined a broader time interval for the fossil calibration point that better reflects uncertainties about the evolutionary history of this group [88]. Comparison of tMRCA estimations between the two studies shows slightly older dates in our analysis, but no significant differences considering the error margins.

Two sources of uncertainty remain concerning the inferred timescale. Firstly, it is now well known that molecular rates are time-dependant [89]–[91], and a single, deep, calibration point at inter-specific level may not provide optimal accuracy for estimations at an intra-specific level. Only a combination of several calibration points at contrasting time scales would be likely to significantly increase dating accuracy [92]–[93]. Unfortunately, for this group we lack both a well-dated Quaternary fossil record or radiocarbon dated specimens from which ancient DNA has been derived. Secondly, our study and that of Robins *et al.* [24] are based only on the mitochondrial genome and thus is subject to intrinsic biases related to maternal inheritance and organelle location [94]–[95]. Information from multiple markers will be needed to derive more robust estimates of the timing of phylogenetic diversification in this recently evolved group.

## Supporting Information

Figure S1 **Result of BEAST analysis using the same parameters as** **Fig. 4** **but using the Yule process speciation model as tree prior and with a single representative of each of the species and mtDNA lineages within the RrC.** Divergence time estimates for labelled nodes A–E are shown in Table S4.
(DOC)

Table S1 **List of haplotypes with GenBank accession numbers and details of collecting locality.**
(DOC)

Table S2 **Details of collecting localities and habitat for all samples listed in Table S1.**
(DOC)

Table S3 **Selection of important zoonotic diseases and pathogens for which members of the** ***Rattus rattus*** **Complex are identified as significant hosts or carriers.**
(DOC)

Table S4 **Comparison of estimated divergence dates from the present study and from Robins et al.** [**28**] **.**
(DOC)

Table S5 **Nucleotide diversity,** ***Pi*** **** [**29**] **for each of the six lineages and two clades of the** ***Rattus rattus*** **Complex, and for various other species of** ***Rattus*** **.**
(DOC)

References S1 **References for Supporting Information.**
(DOC)
