# Supplementary material for: Multiple Geographic Origins of Commensalism and Complex Dispersal History of Black Rats
Source: PLoS One. 2011 Nov 2;6(11):e26357. doi: 10.1371/journal.pone.0026357 (PMC3206810; doi:10.1371/journal.pone.0026357)
Supplement: Figure S1 — Result of BEAST analysis using the same parameters as Fig. 4 but using the Yule process speciation model as tree prior and with a single representative of each of the species and mtDNA lineages within the RrC. Divergence time estimates for labelled nodes A–E are shown in Table S4. (DOC) [file pone.0026357.s001.doc]

Supporting Information for

**Multiple geographic origins of commensalism and complex dispersal history of Black Rats**

Ken P. Aplin*, Hitoshi Suzuki, Alejandro A. Chinen, R. Terry Chesser, José ten Have, Stephen C. Donnellan, Jeremy Austin, Angela Frost, Jean Paul Gonzalez, Vincent Herbreteau, Francois Catzeflis, Julien Soubrier, Yin-Ping Fang, Judith Robins, Elizabeth Matisoo-Smith, Amanda D.S. Bastos, Ibnu Maryanto, Martua H. Sinaga, Christiane Denys, Grace Yap, Ronald A. Van Den Bussche, Chris Conroy, Kevin Rowe, Alan Cooper*

*To whom correspondence should be addressed. E-mail: aplin.ken@gmail.com

**Figure S1**

**
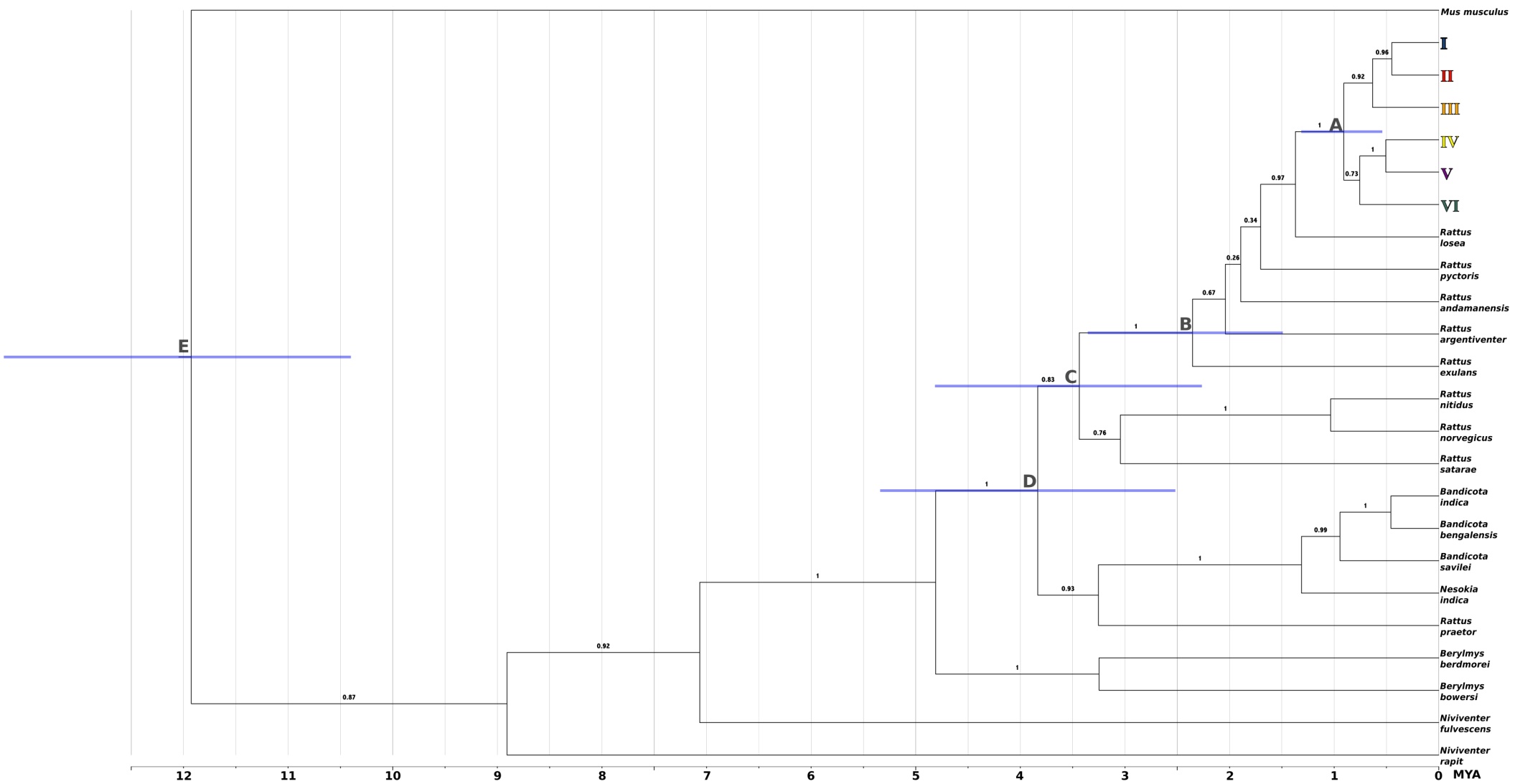
**

**Figure S1. Result of BEAST analysis using the same parameters as Fig. 4 but using the Yule process speciation model as tree prior and with a single representative of each of the species and mtDNA lineages within the RrC.**

Divergence time estimates for labelled nodes A-E are shown in Table S4.
