# Supplementary material for: Multiple Geographic Origins of Commensalism and Complex Dispersal History of Black Rats
Source: PLoS One. 2011 Nov 2;6(11):e26357. doi: 10.1371/journal.pone.0026357 (PMC3206810; doi:10.1371/journal.pone.0026357)
Supplement: Table S1 — List of haplotypes with GenBank accession numbers and details of collecting locality. (DOC) [file pone.0026357.s002.doc]

Supporting Information for

**Multiple geographic origins of commensalism and complex dispersal history of Black Rats**

Ken P. Aplin*, Hitoshi Suzuki, Alejandro A. Chinen, R. Terry Chesser, José ten Have, Stephen C. Donnellan, Jeremy Austin, Angela Frost, Jean Paul Gonzalez, Vincent Herbreteau, Francois Catzeflis, Julien Soubrier, Yin-Ping Fang, Judith Robins, Elizabeth Matisoo-Smith, Amanda D.S. Bastos, Ibnu Maryanto, Martua H. Sinaga, Christiane Denys, Grace Yap, Ronald A. Van Den Bussche, Chris Conroy, Kevin Rowe, Alan Cooper*

*To whom correspondence should be addressed. E-mail: aplin.ken@gmail.com

**Table S1. List of haplotypes with GenBank accession numbers and details of collecting locality.** N refers to the number of individuals observed with a particular haplotype. Additional details of sampling localities are provided in Table S2. Haplotypes identified with an asterisk are shorter segments, typically of 596 bp. All others include a common 945 bp alignment. Haplotypes identified with a dagger symbol were included in the reduced BEAST analysis that used single representatives of each mtDNA lineage with the RrC and each of the other *Rattus* species.

| **Code on trees** | **N** | **Locality No.** | **Taxon** | **RrC Lineage** | **Karyotype** | **Country** | **Accession No** |
| --- | --- | --- | --- | --- | --- | --- | --- |
| 1(Rno-1) | 1 | 1 | *R. norvegicus* | - | - | Denmark | AJ428514 |
| 2(RrI-2) | 1 | 2 | *R. rattus* Complex | I | - | France | JN675517 |
| 3(RrI-3) | 1 | 3 | *R. rattus* Complex | I | - | Senegal | JN675518 |
| 4(RrI-4) | 2 | 4 | *R. rattus* Complex | I | - | Guinea | JN675519 |
| 5(RrII-5) | 1 | 5 | *R. rattus* Complex | II | 42 | South Africa | DQ439819 |
| 6(RrI-6) | 1 | 6 | *R. rattus* Complex | I | - | South Africa | DQ439830 |
| 7(RrI-6) | 1 | 6 | *R. rattus* Complex | I | 38 | South Africa | DQ439833 |
| 8(RrI-6) | 4 | 6 | *R. rattus* Complex | I | - | South Africa | DQ439834 |
| 9(RrI-7) | 1 | 7 | *R. rattus* Complex | I | - | Madagascar | JN675520 |
| 10(RrI-8) | 1 | 8 | *R. rattus* Complex | I | - | Madagascar | JN675521 |
| 11(RrI-9) | 1 | 9 | *R. rattus* Complex | I | - | Madagascar | JN675522 |
| 12(RrI-10) | 1 | 10 | *R. rattus* Complex | I | - | Oman | JN675523 |
| 13(RrI-11) | 1 | 11 | *R. rattus* Complex | I | - | Iran | JN675524 |
| 14(RrIII-12) | 1 | 12 | *R. rattus* Complex | III | - | Pakistan | JN675600 |
| 15(RrIII-12) | 1 | 12 | *R. rattus* Complex | III | - | Pakistan | JN675601 |
| 16(Nin-12) | 1 | 12 | *Nesokia indica* | - | - | Pakistan | AF160605 |
| 17(RrIII-13)* | 1 | 13 | *R. rattus* Complex | III | - | Pakistan | JN675602 |
| 18(RrI-14) | 1 | 14 | *R. rattus* Complex | I | 38 | India | JN675525 |
| 19(RrI-14) | 1 | 14 | *R. rattus* Complex | I | 38 | India | JN675526 |
| 20(RrI-14) | 1 | 14 | *R. rattus* Complex | I | 38 | India | JN675527 |
| 21(RrI-15) | 1 | 15 | *R. rattus* Complex | I | 38 | India | JN675528 |
| 22(RrI-16) | 1 | 16 | *R. rattus* Complex | I | 38 | India | JN675529 |
| 23(RrI-16) | 1 | 16 | *R. rattus* Complex | I | - | India | JN675530 |
| 24(RrI-16) | 1 | 16 | *R. rattus* Complex | I | - | India | JN675531 |
| 25(Rsa-16) | 1 | 16 | *R. satarae* | - | 42 | India | JN675513 |
| 26(RrI-17) | 1 | 17 | *R. rattus* Complex | I | - | India | JN675532 |
| 27(RrI-17) | 1 | 17 | *R. rattus* Complex | I | - | India | JN675533 |
| 28(RrIV-18) | 2 | 18 | *R. rattus* Complex | IV | - | Sri Lanka | JN675603 |
| 29(Rpy-19) | 1 | 19 | *R. pyctoris* | - | - | Nepal | JN675511 |
| 30(Rpy-19) | 1 | 19 | *R. pyctoris* | - | - | Nepal | JN675512 |
| 31(RrIII-19) | 1 | 19 | *R. rattus* Complex | III | - | Nepal | JN675599 |
| 32(Bbe-20) | 1 | 20 | *Bandicota bengalensis* | - | - | Bangladesh | JN675474 |
| 33(RrII-21) | 1 | 21 | *R. rattus* Complex | II | - | Bangladesh | JN675554 |
| 34(RrII-21) | 1 | 21 | *R. rattus* Complex | II | - | Bangladesh | JN675555 |
| 35(RrII-22) | 2 | 22 | *R. rattus* Complex | II | - | Myanmar | JN675556 |
| 36(RrII-23) | 1 | 23 | *R. rattus* Complex | II | - | Myanmar | JN675557 |
| 37(RrII-23) | 2 | 23 | *R. rattus* Complex | II | - | Myanmar | JN675558 |
| 38(RrV-24) | 2 | 24 | *R. rattus* Complex | V | 42 | Thailand | JN675627 |
| 39(RrII-25) | 1 | 25 | *R. rattus* Complex | II | - | Thailand | JN675559 |
| 40(RrII-25) | 1 | 25 | *R. rattus* Complex | II | - | Thailand | JN675560 |
| 41(RrII-26) | 2 | 26 | *R. rattus* Complex | II | - | Thailand | JN675561 |
| 42(Bbr-26) | 1 | 26 | *Berylmys berdmorei* | - | - | Thailand | JN675477 |
| 44(Rex-26) | 1 | 26 | *R. exulans* | - | - | Thailand | JN675496 |
| 45(Bsa-27) | 1 | 27 | *Bandicota savilei* | - | - | Thailand | JN675476 |
| 46(Nfu-28) | 1 | 28 | *Niviventer fulvescens* | - | - | Thailand | JN675479 |
| 47(Bbo-28) | 1 | 28 | *Berylmys bowersi* | - | - | Thailand | JN675478 |
| 48(RrV-28) | 1 | 28 | *R. rattus* Complex | V | - | Thailand | JN675628 |
| 49(RrII-28) | 1 | 28 | *R. rattus* Complex | II | - | Thailand | JN675562 |
| 50(Bin-29) | 1 | 29 | *R. rattus* Complex | - | - | Thailand | JN675475 |
| 51(RrII-30) | 1 | 30 | *R. rattus* Complex | II | - | Laos | JN675563 |
| 52(RrII-30) | 1 | 30 | *R. rattus* Complex | II | - | Laos | JN675564 |
| 53(RrII-30) | 1 | 30 | *R. rattus* Complex | II | - | Laos | JN675565 |
| 54(RrII-30) | 1 | 30 | *R. rattus* Complex | II | - | Laos | JN675566 |
| 55(RrII-30) | 1 | 30 | *R. rattus* Complex | II | - | Laos | JN675567 |
| 56(RrII-31) | 1 | 31 | *R. rattus* Complex | II | - | Laos | JN675568 |
| 57(RrII-31) | 3 | 31 | *R. rattus* Complex | II | - | Laos | JN675569 |
| 58(RrV-32) | 1 | 32 | *R. rattus* Complex | V | - | Laos | JN675625 |
| 59(RrV-32) | 2 | 32 | *R. rattus* Complex | V | - | Laos | JN675626 |
| 60(Rex-32) | 1 | 32 | *R. exulans* | - | - | Laos | JN675497 |
| 61(RrII-33) | 1 | 33 | *R. rattus* Complex | II | - | Laos | JN675570 |
| 62(RrII-33) | 1 | 33 | *R. rattus* Complex | II | - | Laos | JN675571 |
| 63(Rni-33) | 1 | 33 | *R. nitidus* | - | - | Laos | JN675509 |
| 64(RrIV-34) | 1 | 34 | *R. rattus* Complex | IV | - | Laos | JN675604 |
| 65(RrIV-35) | 1 | 35 | *R. rattus* Complex | IV | - | Cambodia | JN675605 |
| 66(Ran-35) | 1 | 35 | *R. andamanensis* | - | - | Cambodia | JN675481 |
| 67(RrIV-36) | 2 | 36 | *R. rattus* Complex | IV | - | Cambodia | JN675606 |
| 68(RrIV-37) | 1 | 37 | *R. rattus* Complex | IV | - | Cambodia | JN675607 |
| 69(RrIV-37) | 1 | 37 | *R. rattus* Complex | IV | - | Cambodia | JN675608 |
| 70(RrIV-37) | 1 | 37 | *R. rattus* Complex | IV | - | Cambodia | JN675609 |
| 71(Rar-38) | 1 | 38 | *R. argentiventer* | - | - | Cambodia | JN675488 |
| 72(Rlo-38) | 1 | 38 | *R. losea* | - | - | Cambodia | JN675499 |
| 73(RrIV-38) | 1 | 38 | *R. rattus* Complex | IV | - | Cambodia | JN675610 |
| 74(RrIV-39) | 1 | 39 | *R. rattus* Complex | IV | - | Vietnam | JN675611 |
| 75(RrIV-39) | 1 | 39 | *R. rattus* Complex | IV | - | Vietnam | JN675612 |
| 76(Rar-40) | 1 | 40 | *R. argentiventer* | - | - | Vietnam | JN675489 |
| 77(Rlo-40) | 1 | 40 | *R. losea* | - | - | Vietnam | JN675500 |
| 78(Rlo-41) | 1 | 41 | *R. losea* | - | - | Vietnam | JN675501 |
| 79(Rar-41) | 1 | 41 | *R. argentiventer* | - | - | Vietnam | JN675490 |
| 80(Rar-41) | 1 | 41 | *R. argentiventer* | - | - | Vietnam | JN675491 |
| 81(Rlo-42) | 1 | 42 | *R. losea* | - | - | Vietnam | JN675502 |
| 82(RrIV-42) | 1 | 42 | *R. rattus* Complex | IV | - | Vietnam | JN675613 |
| 83(RrIV-42) | 1 | 42 | *R. rattus* Complex | IV | - | Vietnam | JN675614 |
| 84(Rlo-42) | 1 | 42 | *R. losea* | - | - | Vietnam | JN675503 |
| 85(Rex-43) | 1 | 43 | *R. exulans* | - | - | Vietnam | JN675498 |
| 86(Rno-43) | 1 | 43 | *R. norvegicus* | - | - | Vietnam | JN675510 |
| 87(RrII-44) | 1 | 44 | *R. rattus* Complex | II | - | Vietnam | JN675572 |
| 88(Ran-44) | 1 | 44 | *R. andamanensis* | - | - | Vietnam | JN675482 |
| 89(Rlo-45) | 1 | 45 | *R. losea* | - | - | Vietnam | JN675504 |
| 90(RrII-45) | 1 | 45 | *R. rattus* Complex | II | - | Vietnam | JN675573 |
| 91(RrII-45) | 1 | 45 | *R. rattus* Complex | II | - | Vietnam | JN675574 |
| 92(Rlo-45) | 1 | 45 | *R. losea* | - | - | Vietnam | JN675505 |
| 93(Rlo-45) | 1 | 45 | *R. losea* | - | - | Vietnam | JN675506 |
| 94(Rlo-45) | 1 | 45 | *R. losea* | - | - | Vietnam | JN675507 |
| 95(Rar-45) | 1 | 45 | *R. argentiventer* | - | - | Vietnam | JN675492 |
| 96(Ran-46) | 1 | 46 | *R. andamanensis* | - | - | China | JN675483 |
| 97(Ran-46) | 1 | 46 | *R. andamanensis* | - | - | China | JN675484 |
| 98(RrII-47) | 1 | 47 | *R. rattus* Complex | II | - | China | JN675575 |
| 99(RrII-47)* | 1 | 47 | *R. rattus* Complex | II | - | China | JN675576 |
| 100(Ran-48) | 1 | 48 | *R. andamanensis* | - | - | China | JN675485 |
| 101(RrII-49) | 1 | 49 | *R. rattus Complex* | II | - | China | JN675577 |
| 102(Ran-50) | 1 | 50 | *R. andamanensis* | - | - | China | JN675486 |
| 103(Ran-50) | 1 | 50 | *R. andamanensis* | - | - | China | JN675487 |
| 104(RrII-50) | 1 | 50 | *R. rattus* Complex | II | - | China | JN675578 |
| 105(RrII-50)* | 1 | 50 | *R. rattus* Complex | II | - | China | JN675579 |
| 106(Nra-51) | 1 | 51 | *Niviventer rapit* | - | - | Indonesia | JN675480 |
| 107(RrIV-52)* | 1 | 52 | *R. rattus* Complex | IV | - | Indonesia | JN675615 |
| 108(RrII-52)* | 1 | 52 | *R. rattus* Complex | II | - | Indonesia | JN675580 |
| 109(Rti-52)* | 1 | 52 | *R. tiomanicus* | VI | - | Indonesia | JN675514 |
| 110(Rti-53) | 3 | 53 | *R. tiomanicus* | VI | - | Indonesia | JN675515 |
| 111(RrII-54)* | 1 | 54 | *R. rattus* Complex | II | - | Indonesia | JN675581 |
| 112(RrII-54)* | 2 | 54 | *R. rattus* Complex | II | - | Indonesia | JN675582 |
| 113(RrII-55) | 1 | 55 | *R. rattus* Complex | II | - | Indonesia | JN675583 |
| 114(RrII-55) | 2 | 55 | *R. rattus* Complex | II | - | Indonesia | JN675584 |
| 115(RrIV-56) | 1 | 56 | *R. rattus* Complex | IV | - | Indonesia | JN675616 |
| 116(RrIV-56) | 2 | 56 | *R. rattus* Complex | IV | - | Indonesia | JN675617 |
| 117(RrIV-57) | 2 | 57 | *R. rattus* Complex | IV | - | Indonesia | JN675618 |
| 118(RrIV-57) | 1 | 57 | *R. rattus* Complex | IV | - | Indonesia | JN675619 |
| 119(Rar-57) | 1 | 57 | *R. argentiventer* | - | - | Indonesia | JN675493 |
| 120(Rar-58) | 1 | 58 | *R. argentiventer* | - | - | Indonesia | JN675494 |
| 121(Rti-58) | 2 | 58 | *R. tiomanicus* | VI | - | Indonesia | JN675516 |
| 122(Rba-59) | 1 | 59 | *R. baluensis* | VI | - | Sabah | JN675495 |
| 123(RrIV-60) | 3 | 60 | *R. rattus* Complex | IV | - | Indonesia | JN675620 |
| 124(RrIV-61) | 1 | 61 | *R. rattus* Complex | IV | - | Indonesia | JN675621 |
| 125(RrIV-61) | 1 | 61 | *R. rattus* Complex | IV | - | Indonesia | JN675622 |
| 126(RrIV-62) | 5 | 62 | *R. rattus* Complex | IV | - | Philippines | JN675623 |
| 127(RrII-62) | 1 | 62 | *R. rattus* Complex | II | - | Philippines | JN675585 |
| 128(RrIV-62) | 1 | 62 | *R. rattus* Complex | IV | - | Philippines | JN675624 |
| 129(RrII-62) | 1 | 62 | *R. rattus* Complex | II | - | Philippines | JN675586 |
| 130(RrII-62) | 1 | 62 | *R. rattus* Complex | II | - | Philippines | JN675587 |
| 131(RrIV-63) | 1 | 63 | *R. rattus* Complex | IV | - | Philippines | DQ191488 |
| 132(Rex-64) | 1 | 64 | *R. exulans* | - | - | Philippines | DQ191486 |
| 133(RrII-65) | 1 | 65 | *R. rattus* Complex | II | - | Taiwan | JN675588 |
| 134(RrII-65) | 1 | 65 | *R. rattus* Complex | II | - | Taiwan | JN675589 |
| 135(RrII-65) | 1 | 65 | *R. rattus* Complex | II | - | Taiwan | JN675590 |
| 136(Rlo-66) | 1 | 66 | *R. losea* | - | - | Taiwan | JN675508 |
| 137(RrII-67) | 2 | 67 | *R. rattus* Complex | II | - | Taiwan | JN675591 |
| 138(RrII-68)* | 3 | 68 | *R. rattus* Complex | II | 42 | Japan | JN675592 |
| 139(RrII-69) | 1 | 69 | *R. rattus* Complex | II | 42 | Japan | JN675593 |
| 140(RrII-70) | 4 | 70 | *R. rattus* Complex | II | 42 | Japan | JN675594 |
| 141(RrI-71) | 5 | 71 | *R. rattus* Complex | I | 38 | Japan | JN675534 |
| 142(RrII-72) | 1 | 72 | *R. rattus* Complex | II | - | Papua New Guinea | JN675595 |
| 143(RrI-73)* | 1 | 73 | *R. rattus* Complex | I | - | Papua New Guinea | JN675535 |
| 144(RrI-74) | 3 | 74 | *R. rattus* Complex | I | - | Australia | JN675536 |
| 145(RrI-75)* | 1 | 75 | *R. rattus* Complex | I | - | NewZealand | JN675537 |
| 146(RrI-76)* | 1 | 76 | *R. rattus* Complex | I | - | Samoa | JN675538 |
| 147(RrI-77)* | 1 | 77 | *R. rattus* Complex | I | - | Society Islands | JN675539 |
| 148(RrI-78)* | 1 | 78 | *R. rattus* Complex | I | - | Society Islands | JN675540 |
| 149(RrI-79) | 1 | 79 | *R. rattus* Complex | I | - | USA | JN675541 |
| 150(RrI-79) | 1 | 79 | *R. rattus* Complex | I | - | USA | JN675542 |
| 151(RrI-80) | 2 | 80 | *R. rattus* Complex | I | - | USA | JN675543 |
| 152(RrII-80) | 1 | 80 | *R. rattus* Complex | II | - | USA | JN675596 |
| 153(RrII-80) | 1 | 80 | *R. rattus* Complex | II | - | USA | JN675597 |
| 154(RrI-80) | 2 | 80 | *R. rattus* Complex | I | - | USA | JN675544 |
| 155(RrII-81) | 1 | 81 | *R. rattus* Complex | II | - | USA | JN675598 |
| 156(RrI-82) | 1 | 82 | *R. rattus* Complex | I | - | USA | JN675545 |
| 157(RrI-83) | 1 | 83 | *R. rattus* Complex | I | - | Brazil | JN675546 |
| 158(RrI-84) | 1 | 84 | *R. rattus* Complex | I | - | Venezuela | JN675547 |
| 159(RrI-85) | 1 | 85 | *R. rattus* Complex | I | - | Guyana | JN675548 |
| 160(RrI-86) | 1 | 86 | *R. rattus* Complex | I | - | Guyana | JN675549 |
| 161(RrI-87) | 1 | 87 | *R. rattus* Complex | I | - | Brazil | JN675550 |
| 162(RrI-87) | 1 | 87 | *R. rattus* Complex | I | - | Brazil | JN675551 |
| 163(Rno-88) | 1 | 88 | *R. norvegicus* | - | - | Wistar Lab | ABO33713 |
| 164(RrI-9a) | 6 | 9a | *R. rattus* Complex | I | - | Madagascar | JN675552 |
| 165(RrI-9b) | 1 | 9b | *R. rattus* Complex | I | - | Madagascar | JN675553 |
