# Supplementary material for: Multiple Geographic Origins of Commensalism and Complex Dispersal History of Black Rats
Source: PLoS One. 2011 Nov 2;6(11):e26357. doi: 10.1371/journal.pone.0026357 (PMC3206810; doi:10.1371/journal.pone.0026357)
Supplement: Table S2 — Details of collecting localities and habitat for all samples listed in Table S1. (DOC) [file pone.0026357.s003.doc]

Supporting Information for

**Multiple geographic origins of commensalism and complex dispersal history of Black Rats**

Ken P. Aplin*, Hitoshi Suzuki, Alejandro A. Chinen, R. Terry Chesser, José ten Have, Stephen C. Donnellan, Jeremy Austin, Angela Frost, Jean Paul Gonzalez, Vincent Herbreteau, Francois Catzeflis, Julien Soubrier, Yin-Ping Fang, Judith Robins, Elizabeth Matisoo-Smith, Amanda D.S. Bastos, Ibnu Maryanto, Martua H. Sinaga, Christiane Denys, Grace Yap, Ronald A. Van Den Bussche, Chris Conroy, Kevin Rowe, Alan Cooper*

*To whom correspondence should be addressed. E-mail: aplin.ken@gmail.com

**Table S2**. Details of collecting localities and habitat for all samples listed in Table S1. Latitude and longitudes are expressed as decimals with negative values for latitude indicating locations in the southern hemisphere. Habitat codes are: F: forest; R: rural; U: urban; dash: unknown.

| Local No. | Locality | Habitat | Country | Latitude | Longitude | Collector |
| --- | --- | --- | --- | --- | --- | --- |
| 1 | Copenhagen | - | Denmark | 55.683 | 12.583 |  |
| 2 | Corsica | - | France | 42.000 | 9.000 | C. Denys |
| 3 | Kedougou | - | Senegal | 12.567 | 12.217 | C. Denys |
| 4 | Yufraya | - | Guinea | 11.500 | 14.750 | C. Denys |
| 5 | Giyani, Limpopo Province | - | South Africa | -23.416 | 30.786 | L. Mabunda |
| 6 | Hammanskraal, Gauteng Province | - | South Africa | -25.371 | 28.188 | M. Matjila |
| 7 | Amphijoroa | - | Madagascar | -16.230 | 46.470 | F. Catzeflis |
| 8 | Tapera | - | Madagascar | -24.900 | 47.130 | F. Catzeflis |
| 9 | Betsizaraina | - | Madagascar | -19.950 | 48.750 | F. Catzeflis |
| 9a | Ankaratra | - | Madagascar | -19.417 | 47.200 | J. Patton |
| 9b | Ankaratra | - | Madagascar | -19.417 | 47.200 | J. Patton |
| 9c | Ambatolahy | - | Madagascar | -22.417 | 44.283 | J. Patton |
| 9d | Ronomafankely | - | Madagascar | ? | ? | J. Patton |
| 10 | Dhofar | - | Oman | 17.033 | 54.167 |  |
| 11 | Bandar-e Hamiran, Hormozgan Prov. | - | Iran | 26.750 | 55.667 |  |
| 12 | Islamabad | - | Pakistan | 33.700 | 73.133 | F. Catzeflis |
| 13 | Gujarkhan Sang [56 km from Islamabad] | - | Pakistan | 33.800 | 73.200 |  |
| 14 | Mudumalai | - | India | 11.620 | 76.570 | F. Catzeflis |
| 15 | Ooty | - | India | 11.400 | 76.700 | F. Catzeflis |
| 16 | Kotagiri | - | India | 11.430 | 76.880 | F. Catzeflis |
| 17 | Attur | - | India | 11.600 | 78.617 | F. Catzeflis |
| 18 | Central highlands | - | Sri Lanka | 7.000 | 81.000 | T. H. Yosida |
| 19 | Kathmandu | - | Nepal | 27.700 | 85.300 |  |
| 20 | Gazipur, Dhaka | U | Bangladesh | 23.717 | 90.400 | K. Aplin |
| 21 | Feni | U | Bangladesh | 23.000 | 91.400 | K. Aplin |
| 22 | Yangon | R | Myanmar | 16.817 | 96.133 | K. Aplin |
| 23 | Yezin | R | Myanmar | 19.750 | 96.250 | K. Aplin |
| 24 | Chaiyaphum | - | Thailand | 15.500 | 102.100 | T. H. Yosida |
| 25 | Chulaphorn Dam | - | Thailand | 16.500 | 101.600 | C. Denys |
| 26 | Huaymalai, Sangkhlaburi, Kanchanaburi | - | Thailand | 13.983 | 99.550 | V. Herbreteau |
| 27 | Ban Wiang, Rong Kwang, Phrae | R | Thailand | 18.117 | 100.200 | K. Aplin |
| 28 | Nam Man, Muang, Loei | - | Thailand | 17.517 | 101.617 | V. Herbreteau |
| 29 | Chalerm Prakiat, Nakhon Ratchasima | - | Thailand | 14.950 | 102.133 | V. Herbreteau |
| 30 | Luang Namtha | R | Laos | 21.050 | 101.483 | K. Aplin |
| 31 | Houay Khot | R,F | Laos | 19.967 | 102.167 | K. Aplin |
| 32 | Vientianne | R | Laos | 17.967 | 102.617 | K. Aplin |
| 33 | Houaphanh | R,F | Laos | 20.417 | 104.033 | K. Aplin |
| 34 | Lamam, Sekong Province | R | Laos | 15.350 | 106.717 | K. Aplin |
| 35 | Cardamom Mts | F | Cambodia | 12.067 | 103.317 | A. Olsson |
| 36 | Speu, Kampong Cham Province | R | Cambodia | 11.383 | 104.567 | A. Frost |
| 37 | Cambodian Agr. Res. & Devel. Inst. Phnom Penh | R | Cambodia | 11.550 | 104.950 | A. Frost |
| 38 | Somrong Commune, Kampong Cham Province | R | Cambodia | 11.983 | 105.433 | A. Frost |
| 39 | Tien Giang Province | R | Vietnam | 10.017 | 105.767 | K. Aplin |
| 40 | Soc Trang Province | R | Vietnam | 9.567 | 106.000 | K. Aplin |
| 41 | Cu Chi, Ho Chi Minh City Province | R | Vietnam | 10.950 | 106.650 | K. Aplin |
| 42 | Near HCM City | - | Vietnam | 10.750 | 106.700 |  |
| 43 | Bac Binh, Binh Thuan Province | R | Vietnam | 10.917 | 108.100 | K. Aplin |
| 44 | Nth Vietnam | - | Vietnam | 21.000 | 105.500 |  |
| 45 | Vin Phuc Province | R | Vietnam | 21.017 | 105.850 | K. Aplin |
| 46 | Area No. 2, 1 km E Menglung, Yunnan | - | China | 21.917 | 101.267 |  |
| 46 | Area No. 2, near Menglung, Yunnan | - | China | 21.917 | 101.267 |  |
| 47 | Yunnan Institute of Tropical Botany | - | China | 21.917 | 101.267 |  |
| 48 | Kunming Experimental Station, Menglung, Yunnan | - | China | 21.883 | 101.300 |  |
| 49 | Tongzi | - | China | 29.567 | 107.950 |  |
| 50 | Hong Kong | U | China | 22.267 | 114.183 | K. Chung |
| 51 | Ketambe Research Station, Sumatra | - | Indonesia | 3.683 | 97.650 |  |
| 52 | Jakarta | - | Indonesia | -6.180 | 106.830 |  |
| 53 | Pangandaran Nature Reserve, Ciamis, West Java | - | Indonesia | -7.167 | 108.667 |  |
| 54 | Yogyakarta, Indonesia | - | Indonesia | -7.833 | 110.367 |  |
| 55 | Bali | - | Indonesia | -8.390 | 115.217 |  |
| 56 | Lombok | - | Indonesia | -8.500 | 116.333 |  |
| 57 | Sumbawa | - | Indonesia | -8.500 | 118.000 |  |
| 58 | Central Kalimantan | - | Indonesia | -2.000 | 114.000 |  |
| 59 | Mt Kinabalu | F | Sabah | 6.067 | 116.550 | M. Ruedi |
| 60 | Manado, Sulawesi | - | Indonesia | 1.550 | 124.867 |  |
| 61 | Flores | - | Indonesia | -8.700 | 121.000 |  |
| 62 | Los Banos | R | Philippines | 14.180 | 121.150 | K. Aplin |
| 63 | Luzon | - | Philippines | 15.000 | 122.000 |  |
| 64 | Luzon | - | Philippines | 16.000 | 122.000 |  |
| 65 | Taichung City | - | Taiwan | 24.149 | 120.684 |  |
| 65a | Shou-Feng, Hualien | - | Taiwan | 24.117 | 120.467 |  |
| 65b | Shindian City, Taipei | - | Taiwan | 25.039 | 121.525 |  |
| 66 | Unknown | - | Taiwan | 24.000 | 121.000 |  |
| 67 | Lanyu (Orchid Island), Taitung | - | Taiwan | 22.017 | 121.567 |  |
| 68 | Amamioshima, Ryukyu Islands | - | Japan | 28.317 | 129.433 |  |
| 69 | Mizayaki, Kyushu | U | Japan | 35.683 | 139.800 | A. Chinen |
| 70 | Tokyo, Honshu | U | Japan | 35.683 | 139.800 | A. Chinen |
| 71 | Otaru, Hokkaido | U | Japan | 43.200 | 140.983 | A. Chinen |
| 72 | Passam Agricultural Station, 8 mi S Wewak | R | Papua New Guinea | -3.695 | 143.633 |  |
| 73 | Sideia Is, Milne Bay Province | R | Papua New Guinea | -10.550 | 150.850 |  |
| 74 | Sydney | U | Australia | -33.900 | 151.183 | K. Aplin |
| 75 | Titirangi, New Zealand | - | NewZealand | -36.951 | 174.651 |  |
| 76 | Samoa | - | Samoa | -13.600 | 197.550 |  |
| 77 | Raiatea | - | Society Islands | -16.820 | 208.570 |  |
| 78 | Huahine | - | Society Islands | -16.720 | 208.900 |  |
| 79 | Berkeley, California | U | USA | 37.872 | 237.743 |  |
| 79a | San Lorenzo, California | U | USA | 37.677 | 237.884 |  |
| 80 | Oakland, California | U | USA | 37.770 | 237.780 |  |
| 81 | Orinda, Contra Costa County | U | USA | 37.872 | 237.825 |  |
| 82 | Peaslee Creek, Stanislaus County | U | USA | 37.622 | 239.473 |  |
| 83 | Acre, Sobral, left bank Rio Juru· | - | Brazil | -8.367 | 287.183 |  |
| 84 | Bolivar | - | Venezuela | 10.000 | 293.000 | F. Catzeflis |
| 85 | Cayene | - | Guyana | 4.883 | 307.650 | F. Catzeflis |
| 86 | Piste Nacibo | - | Guyana | 4.883 | 307.650 | F. Catzeflis |
| 87 | Bahia, Ilha das Fontes, 36 km NW Salvador | - | Brazil | -12.673 | 321.363 |  |
