# Supplementary material for: Multiple Geographic Origins of Commensalism and Complex Dispersal History of Black Rats
Source: PLoS One. 2011 Nov 2;6(11):e26357. doi: 10.1371/journal.pone.0026357 (PMC3206810; doi:10.1371/journal.pone.0026357)
Supplement: Table S3 — Selection of important zoonotic diseases and pathogens for which members of the Rattus rattus Complex are identified as significant hosts or carriers. (DOC) [file pone.0026357.s004.doc]

Supporting Information for

**Multiple geographic origins of commensalism and complex dispersal history of Black Rats**

Ken P. Aplin*, Hitoshi Suzuki, Alejandro A. Chinen, R. Terry Chesser, José ten Have, Stephen C. Donnellan, Jeremy Austin, Angela Frost, Jean Paul Gonzalez, Vincent Herbreteau, Francois Catzeflis, Julien Soubrier, Yin-Ping Fang, Judith Robins, Elizabeth Matisoo-Smith, Amanda D.S. Bastos, Ibnu Maryanto, Martua H. Sinaga, Christiane Denys, Grace Yap, Ronald A. Van Den Bussche, Chris Conroy, Kevin Rowe, Alan Cooper*

*To whom correspondence should be addressed. E-mail: aplin.ken@gmail.com

**Table S3**. Selection of important zoonotic diseases and pathogens for which members of the *Rattus rattus* Complex are identified as significant hosts or carriers. Note that many of the diseases are reported either in Asian populations or in those colonised by ‘ship rats’, derivatives of Lineage I of the RrC, with a smaller number having a pan-global distribution.

| **Zoonotic Disease OR PATHOGEN** | **Asia** | **Non-Asian** |
| --- | --- | --- |
| Plague (*Yersinia pestis*) | Myanmar, Cambodia^1^ | Madagascar^2^, USA^1^ |
| Seoul Virus (Bunyaviridae) | Thailand^3^, Cambodia^4^ | USA^5,6^ |
| Leptospirosis (*Leptospira*) | Andaman Islands^7^, Korea^8^ | New Zealand^9^, Zimbabwe^10^ |
| Bartonellosis (*Bartonella* spp.) | China^11^ | United States & Portugal^12^ |
| *Angiostrongylus cantonensis* |  | Carribean13,14 |
| Fasciolosis (*Fasciola hepatica*) |  | France^15^ |
| Trypanosomiasis (*Trypanosoma* *lewisi*) |  | Madagascar^16^ |
| Chagas Disease (*Trypanosoma* *cruzi*) |  | Venezuela^17^ |
| Leishmaniasis (*Leishmania infantum*) |  | Italy, Iraq, Oman^18^ |
| Rift Valley fever virus (Bunyaviridae) |  | Senegal^19^ |
| West Nile encephalitis (Flaviviridae) |  | USA^20^ |
| Salmonelloses (*Salmonella*) | Malaysia^21^ |  |
| *Schistosoma incognitum* | Thailand^22^ |  |
| *Schistosoma japonicum* | Philippines^23^ |  |
| Flea-borne Spotted Fever (*Rickettsia felis*) | Indonesia^24^ |  |
| Spotted fever (*Rickettsia honei*) | Thailand^25^ |  |
| Murine Typhus (*Rickettsia* *typhi*) | Myanmar, Nepal^26^ |  |
| Indian Tick Typhus (*Rickettsia conori*) | India^27^ |  |
