# Supplementary material for: Multiple Geographic Origins of Commensalism and Complex Dispersal History of Black Rats
Source: PLoS One. 2011 Nov 2;6(11):e26357. doi: 10.1371/journal.pone.0026357 (PMC3206810; doi:10.1371/journal.pone.0026357)
Supplement: Table S4 — Comparison of estimated divergence dates from the present study and from Robins et al. [28] . (DOC) [file pone.0026357.s005.doc]

Supporting Information for

**Multiple geographic origins of commensalism and complex dispersal history of Black Rats**

Ken P. Aplin*, Hitoshi Suzuki, Alejandro A. Chinen, R. Terry Chesser, José ten Have, Stephen C. Donnellan, Jeremy Austin, Angela Frost, Jean Paul Gonzalez, Vincent Herbreteau, Francois Catzeflis, Julien Soubrier, Yin-Ping Fang, Judith Robins, Elizabeth Matisoo-Smith, Amanda D.S. Bastos, Ibnu Maryanto, Martua H. Sinaga, Christiane Denys, Grace Yap, Ronald A. Van Den Bussche, Chris Conroy, Kevin Rowe, Alan Cooper*

*To whom correspondence should be addressed. E-mail: aplin.ken@gmail.com

**Table S4.** Comparison of estimated divergence dates from the present study and from Robins et al. [28].

|  | **A1** | | | **A2** | | | **A3** | | |
| --- | --- | --- | --- | --- | --- | --- | --- | --- | --- |
| **Clade/Taxon** | **tMRCA estimation** | **95% HPD lower** | **95% HPD upper** | **tMRCA estimation** | **95% HPD lower** | **95% HPD upper** | **tMRCA estimation** | **95% HPD lower** | **95% HPD upper** |
| **RcR LI** | 163,820 | 85,839 | 250,420 |  |  |  |  |  |  |
| **RcR LII** | 229,440 | 131,090 | 343,850 |  |  |  |  |  |  |
| **RcR LIII** | 205,840 | 94,239 | 328,710 |  |  |  |  |  |  |
| **RcR LIV** | 238,640 | 130,550 | 362,080 |  |  |  |  |  |  |
| **RcR LV** | 55,122 | 14,396 | 106,000 |  |  |  |  |  |  |
| **RcR LVI** | 335,840 | 167,990 | 515,170 |  |  |  |  |  |  |
| **Ran** | 272,830 | 135,600 | 431,970 |  |  |  |  |  |  |
| **Rar** | 210,210 | 104,440 | 329,520 |  |  |  |  |  |  |
| **Rex** | 288,180 | 143,600 | 455,320 |  |  |  |  |  |  |
| **Rlo** | 386,680 | 216,580 | 577,000 |  |  |  |  |  |  |
| **Rni** | 224,970 | 89,369 | 375,060 |  |  |  |  |  |  |
| **Rno** | 169,460 | 78,419 | 276,190 |  |  |  |  |  |  |
| **Rpy** | 22,162 | 199 | 55,486 |  |  |  |  |  |  |
| **Mus** | 457,720 | 251,750 | 690,620 |  |  |  |  |  |  |
| **A** | 1,015,000 | 639,160 | 1,421,000 | 907,520 | 539,440 | 1,314,600 |  |  |  |
| **B** | 2,341,700 | 1,467,600 | 3,252,300 | 2,313,800 | 1,462,100 | 3,341,600 | 2,170,000 | 920,000 | 3,850,000 |
| **C** | 3,527,700 | 2,278,000 | 4,889,500 | 3,494,000 | 2,228,300 | 4,922,200 | 2,900,000 | 1,440,000 | 5,140,000 |
| **D** | 3,827,000 | 2,506,400 | 5,233,400 | 3,837,400 | 2,495,300 | 5,325,600 | 3,510,000 | 1,790,000 | 6,970,000 |
| **E** | 12,031,000 | 10,400,000 | 13,763,000 | 11,926,000 | 10,400,000 | 13,717,000 | 11,620,000 | 10,940,000 | 12,270,000 |

The rows labelled A-E correspond to annotated nodes on the timed phylogenetic trees shown in Figs 3, S1.

Columns A1: BEAST analysis on the entire dataset using 10.4/14 MYA as calibration point for *Mus*/*Rattus* split; HKY + G + I as model of nucleotide substitution; coalescent constant population size as tree prior - 30,000,000 iteration.

Columns A2: BEAST analysis on one representative per clade using 10.4/14 MYA as calibration point for Mus/Rattus split - HKY + G + I as model of nucleotide substitution; Yule speciation process as tree prior; 30,000,000 iterations.

Columns A3: A3. Robins et al. [28] BEAST analysis on complete mitochondrial genomes using 11/12.3MYA; GTR + G4 + I as model of nucleotide substitution; 5,000,000 iterations.
