# Supplementary material for: Multiple Geographic Origins of Commensalism and Complex Dispersal History of Black Rats
Source: PLoS One. 2011 Nov 2;6(11):e26357. doi: 10.1371/journal.pone.0026357 (PMC3206810; doi:10.1371/journal.pone.0026357)
Supplement: Table S5 — Nucleotide diversity, Pi [29] for each of the six lineages and two clades of the Rattus rattus Complex, and for various other species of Rattus . (DOC) [file pone.0026357.s006.doc]

Supporting Information for

**Multiple geographic origins of commensalism and complex dispersal history of Black Rats**

Ken P. Aplin*, Hitoshi Suzuki, Alejandro A. Chinen, R. Terry Chesser, José ten Have, Stephen C. Donnellan, Jeremy Austin, Angela Frost, Jean Paul Gonzalez, Vincent Herbreteau, Francois Catzeflis, Julien Soubrier, Yin-Ping Fang, Judith Robins, Elizabeth Matisoo-Smith, Amanda D.S. Bastos, Ibnu Maryanto, Martua H. Sinaga, Christiane Denys, Grace Yap, Ronald A. Van Den Bussche, Chris Conroy, Kevin Rowe, Alan Cooper*

*To whom correspondence should be addressed. E-mail: aplin.ken@gmail.com

**Table S5.** Nucleotide diversity, *Pi* [29] for each of the six lineages and two clades of the *Rattus rattus* Complex, and for various other species of *Rattus*.

|  | **N** | ***Pi*** |
| --- | --- | --- |
| *R. rattus* Lineage I | 35 | 0.0052 ± 0.0005 |
| *R. rattus* Lineage II | 40 | 0.0084 ± 0.0006 |
| *R. rattus* Lineage III | 3 | 0.0120 ± 0.0000 |
| *R. rattus* Lineage IV | 22 | 0.0084 ± 0.0009 |
| *R. rattus* Lineage V | 4 | 0.0025 ± 0.0006 |
| *R. rattus* Lineage VI | 3 | 0.0162 ± 0.0046 |
|  |  |  |
| Clade A of *R. rattus* Complex | 78 | 0.0225 ± 0.0011 |
| Clade B of *R. rattus* Complex | 29 | 0.0209 ± 0.0033 |
| All of *R. rattus* Complex | 107 | 0.0358 ± 0.0014 |
|  |  |  |
| *R. losea* | 10 | 0.0189 ± 0.0020 |
| *R. argentiventer* | 7 | 0.0108 ± 0.0017 |
| *R. exulans* | 4 | 0.0115 ± 0.0048 |
| *R andamanensis* | 7 | 0.0095 ± 0.0021 |

N = number of distinct haplotypes.
