# Supplementary material for: Multiple Geographic Origins of Commensalism and Complex Dispersal History of Black Rats
Source: PLoS One. 2011 Nov 2;6(11):e26357. doi: 10.1371/journal.pone.0026357 (PMC3206810; doi:10.1371/journal.pone.0026357)
Supplement: References S1 — References for Supporting Information. (DOC) [file pone.0026357.s007.doc]

Supporting Information for

**Multiple geographic origins of commensalism and complex dispersal history of Black Rats**

Ken P. Aplin*, Hitoshi Suzuki, Alejandro A. Chinen, R. Terry Chesser, José ten Have, Stephen C. Donnellan, Jeremy Austin, Angela Frost, Jean Paul Gonzalez, Vincent Herbreteau, Francois Catzeflis, Julien Soubrier, Yin-Ping Fang, Judith Robins, Elizabeth Matisoo-Smith, Amanda D.S. Bastos, Ibnu Maryanto, Martua H. Sinaga, Christiane Denys, Grace Yap, Ronald A. Van Den Bussche, Chris Conroy, Kevin Rowe, Alan Cooper*

*To whom correspondence should be addressed. E-mail: aplin.ken@gmail.com

**References for Supporting Information**

1. G. I. Twigg (1978) *Mamm. Rev.* **8***,* 77.
2. J. M. Duplantier, J. B. Duchemin, S. Chanteau, E. Carniel (2005) *Vet. Res*. **36**, 437.
3. Y. Suputthamongkol, *et al*., (2005) *S. E. Asian J. Trop. Med. Publ. Health* **36**, 700.
4. J. M. Reynes *et al*. (2003) *Microbes Infect*. **5**, 769.
5. G. E. Glass *et al*. (1998) *Amer. J. Trop. Med. Hyg*. **59**, 699.
6. Y. S. Kim *et al*. *Nephron* **71**, 419.
7. S. Shama, P. Vijayachari, A. P. Sugunan, K. Jataranaseenivasan, S. C. Shegal (2006) *Am. J. Trop. Med. Hyg*. **74**, 278.
8. B. M. Rim, C. W. Rim, W. H. Chang, Kakoma, I. (1993) *J.* *Wildl. Dis*. **29**, 602.
9. S. C. Hathaway, D. K. Blakemore (1981) *J. Hyg. Camb.* **87**, 427.
10. J. M. Dalu, S. A. Feresu (1997) *Belg. J. Zool*. **127**, 105.
11. D. M. Li, D. Z. Yu, Q. Y. Liu, Z. D. Gong (2004) *Zhonghua Liu Xing Bing Xue Za Zhi* **25**, 934.
12. B. A. Ellis *et al*. (1999) *J. Infect.Dis*. **180**, 220.
13. C. P. Raccurt, J. Blaise, M. C. Durette-Desset (2003) *Trop. Med. Int. Health* **8**, 423.
14. J. F. Lindo *et al*. (2002) *Emerg. Infect. Dis*. **8**, 324.
15. E. Magnanou, R, Fons, C. Feliu, S. Morand (2006) *Parasitol. Res.* **99**, 97.
16. J. Laakkonen, J. T. Lehtonen, H. Ramiarinjanahary, P.C. Wright (2003) In *ACIAR Monograph No. 96.* *Rats, Mice and People: Rodent Biology and Management*, G. R. Singleton, L. A. Hinds, C. J. Krebs, D. M. Spratt, (Eds) (Australian Centre Intern. Agric. Res., Canberra), pp. 37-40..
17. L. Herrera, S. Urdaneta-Morales (1997) *Rev. Inst. Med. Trop. Sao Paulo* **39**, 279.
18. P. Desjeux (1991) Information of epidemiology and control of the leishmaniases by country or territory. World Health Organization: Geneva, WHO/ LEISH/91.30.
19. D. Gora *et al*. (2000) *Microbes Infect*. **2**, 343.
20. G. Dietrich *et al*. (2005) *Vect. Borne Zoonot. Dis*. **5**, 288.
21. P. G. Joseph, H. T. Yee, S. P. Sivanandan (1984) *S. Asian J. Trop. Med. Publ. Health* **15**, 326.
22. T. Bunnag, S. Thirachandra, P. Impand, P. Vorasanta, S. Imlarp (1983) *S. Asian J. Trop. Med. Publ. Health* **14**, 163
23. L. M. Fedorko (1999) *S. Asian J. Trop. Med. Publ. Health* **30**, 343.
24. J. Jiang *et al*. (2006) *Emerg. Infect. Dis*. **12,** 1281.
25. J. Jiang *et al*. (2005) *Emerg. Infect. Dis*. **9**, 1473.
26. A. F.Azad, C. B. Beard (1998) *Emerg. Infect. Dis*. **4**, 180.
27. V. S. Padbidri *et al*. (1984) *Intern. J. Zoonoses* **11**, 45.
28. J. H. Robins *et al.* (2008) *Molec. Phylogenet. Evol.* **49**, 460.
29. M. Nei, S. Kumar (2000) *Molecular Evolution and Phylogenetics*. Oxford Univ. Press: New York.
